# Effects of the improved application of *Bacillus halotolerans* on the microbial community and volatile components of high-temperature daqu

**DOI:** 10.3389/fmicb.2025.1626160

**Published:** 2025-06-27

**Authors:** Wei Cheng, Lele Guo, Xijia Xue, Chao Jiang, Qiang Chang, Xuefeng Chen

**Affiliations:** ^1^School of Biology and Food Engineering, Fuyang Normal University, Fuyang, China; ^2^School of Food Science and Engineering, Shaanxi University of Science and Technology, Xi'an, China; ^3^College of Urban and Rural Construction, Fuyang Institute of Technology, Fuyang, China; ^4^Anhui WenWang Brewery Co., Ltd., Fuyang, China

**Keywords:** high-temperature daqu (HTD), fortification, microbial community, *Bacillus*, volatile components (VOCs)

## Abstract

**Introduction:**

Studies found similar dynamics in the physicochemical properties, microbial communities, and flavor compounds during fortified daqu fermentation. However, there have been few studies on the application of *B. halotolerans* to fortified high-temperature daqu (HTD), and its effects on the physicochemical parameters, microbial communities, and volatile components (VOCs) of fortified HTD are unclear.

**Methods:**

During the fermentation of *B. halotolerans* fortified HTD, the amplicon sequencing was used to analyze the microbial community, headspace solid-phase microextraction-gas chromatography–mass spectrometry (HS-SPME-GC–MS) was used to analyze the VOCs, and the relationships between physicochemical parameters, dominant microbial communities, and VOCs were analyzed based on redundancy analysis (RDA).

**Results:**

After fortification with *B. halotolerans*, the acidity, saccharification power, and fermentation power of fortified HTD were higher than those of traditional HTD. The fortification with *B. halotolerans* had a significant effect on bacterial diversity, with an increase in the relative abundance of Bacillu. For fortified HTD, the contents of certain VOCs, such as alcohols and esters, were improved compared to their contents in traditional HTD at the end of daqu fermentation. Further, after fortification with *B. halotolerans*, the effects of physicochemical properties on the composition and function of bacterial flora were greater than those on fungal flora, while fungal flora had a greater impact than bacterial flora on VOCs.

**Discussion:**

The fortification with *B. halotolerans* controlled microbial metabolism by altering the composition and abundance of certain microorganisms and promoting the production of specific VOCs, which also influenced the physicochemical properties of HTD. These results provide a basis and new insights for the application of functional strains in daqu fermentation.

## 1 Introduction

Baijiu, a traditional distilled spirit, has represented Chinese culture and tradition for over 1,000 years (Cheng et al., 2023a). Baijiu is often produced from cereals using solid-state fermentation and saccharifying starters known as jiuqu (such as daqu, xiaoqu, and fuqu), and the technology and processes for producing baijiu are complex (Cheng et al., 2022; Zheng and Han, 2016). The flavor is a vital quality marker of baijiu and is influenced by raw materials, saccharifying starters, fermentation processes, storage, and maturation (Cheng et al., 2022; Fan et al., 2018). Daqu serves as a fermentation starter and contains raw materials for baijiu fermentation, including microorganisms and their metabolic enzymes, as well as flavor compounds and their precursors. The flavor compounds are the vital factors influencing the aroma of baijiu (Fan et al., 2018; Fu et al., 2023; He et al., 2019a). Based on the maximum culture temperature, daqu can be classified as high-temperature daqu (HTD), medium-temperature daqu (MTD), or low-temperature daqu (LTD) (Zheng and Han, 2016). In addition, *Bacillus* species play a crucial role in determining the flavor of various types of daqu, and other microorganisms can interact with the secondary metabolites of *Bacillus*, thereby maintaining the relative stability of the daqu microecosystem (He et al., 2019b; He et al., 2020; Tong et al., 2022).

Due to the significant influence of materials, environment, and fermentation time, the microbial types and contents of daqu are complex, and the metabolic activity of microbes significantly affects the flavor of baijiu (Cheng et al., 2023b). Microorganisms with special functions have been isolated from daqu (Cheng et al., 2023b; He et al., 2019b; Li et al., 2017; Wang P. et al., 2017), and the application of these selected strains increased the types and contents of enzymes and VOCs in daqu, which may affect the diversity of Indigenous microbial communities (Li et al., 2017; Wang P. et al., 2017). In particular, *Bacillus* species can secrete enzymes that are vital for regulating the microbiome and producing flavor compounds in daqu (He et al., 2019b; Li et al., 2017; Wang P. et al., 2017; Yang et al., 2022). For example, bioaugmentation with *B. velezensis* and *B. subtilis* alters the microbial community and metabolic activity, further improving the flavor of daqu (He et al., 2019b). Fortification with *B. licheniformis* alters the metabolic activity of the Indigenous microflora in daqu, leading to an increase in the concentrations of aromatic compounds (Wang P. et al., 2017). The composition and metabolic activity of the microbial community in daqu may be influenced by the presence of *B. licheniformis* (Li et al., 2017). In summary, these studies found similar dynamics in the physicochemical properties, microbial communities, and flavor compounds during fortified daqu fermentation. However, there have been few studies on the application of *B. halotolerans* to fortified HTD, and its effects on the physicochemical parameters, microbial communities, and VOCs of HTD are unclear.

Meanwhile, the contribution of the microbial community to VOCs at different fermentation stages of daqu is unclear, especially the effect of fortification strains on the VOCs and quality of fortified daqu (He et al., 2019b; Li et al., 2017; Xu B. et al., 2022; Yang et al., 2022). In addition, the species of *Bacillus* in the HTD are ecological generalists, and their roles in micro-ecosystems remain unclear (Zhang et al., 2022a; Liu et al., 2025; Tu et al., 2024; Zhang et al., 2022b). In the present study, the physicochemical parameters, microbial communities, and VOCs of HTD were evaluated, and correlation analyses were performed during the fermentation process of HTD bioaugmentation with *B. halotolerans*. These findings revealed the changes and contributions of microorganisms to the formation of VOCs in fortified HTD, which will help improve the quality of HTD through bioaugmentation.

## 2 Materials and methods

### 2.1 *Bacillus halotolerans*, fortified HTD manufacturing, and sample collection

*Bacillus halotolerans* was isolated from liangpei (cooked grains) after stacking fermentation, mixed with jiuqu, and subjected to high-temperature stacking fermentation for 36–48 h (Yan et al., 2013; Cheng et al., 2022). *B. halotolerans* was stored at the General Microbial Culture Preservation Management Center of China (CGMCC no. 28161). Fortified HTD was prepared by exogenously inoculating *B. halotolerans* based on the manufacturing processes of traditional HTD. Solutions containing *B. halotolerans* were added and mixed with raw materials before shaping the fortified HTD at a concentration of 1 × 10^6^ colony-forming units/mL (Xu B. et al., 2022; Zhang P. et al., 2024; Zhang Y. et al., 2024; He et al., 2019a), accounting for 5% of the total mass of the raw materials. *B. halotolerans* is typically cultured as a liquid and then introduced into raw materials at a mass ratio of 5–10% (Cheng et al., 2022). The mixture was shaped into bricks and placed in a fermentation room (Qu room) for spontaneous fermentation. Additionally, traditional HTD was manufactured without inoculating the strain, using the same process.

Fortified HTD (cultured for 65 d) was cultivated in a distillery (N32°89′, E115°81′) in Anhui Province, China, and was sampled from July to September 2024, according to a previously introduced method (Fan et al., 2018). Samples were collected during the fermentation process and manually separated to avoid heat-induced damage to microorganisms and VOCs during mechanical crushing (Fan et al., 2018).

Samples of traditional HTD (CD group) and fortified HTD (CS group) were divided into two parts. One portion (500 g) of the samples, which was used for determining the physicochemical properties, enzyme activities, and VOC levels, was stored at 4°C. Another portion (100 g) of the samples, which was used for subsequent DNA extraction, was kept at −80°C.

### 2.2 Determination of the physicochemical properties and enzyme activities

The physicochemical properties of the samples were assessed following the national standard method QB/T 4257–2011 (QB, People’s Republic of China Professional Standard, 2011). Specifically, moisture content was determined by drying the daqu sample at 105°C until a constant weight was achieved. Starch molecules were hydrolyzed into reducing sugars using hydrochloric acid, and the reducing sugar content was calculated based on the colorimetric titration reaction between reducing sugars and Ferling’s solution. The starch content was calculated based on the amount of reducing sugar produced. Acidity was determined via acid–base titration. Saccharification power was assessed by measuring the milligrams of glucose produced per hour from the enzymatic conversion of soluble starch by 1.0 g of absolutely dry daqu at 35°C and pH 4.6. Fermentation power was evaluated by fermenting 0.5 g of absolutely dry daqu sample at 30°C for 72 h and measuring the volume of CO2 produced during the fermentation process. All measurements were conducted in triplicate, and the results are expressed as the mean values (Cheng et al., 2023a).

### 2.3 DNA extraction, PCR amplification, and sequencing

#### 2.3.1 DNA extraction and PCR amplification

According to the manufacturer’s instructions and a previously described method (Zhang et al., 2014), an E. Z. N. A. Soil DNA Kit (Omega Biotek, Norcross, GA, USA) was used to extract genomic DNA from the samples. For the bacterial *16S rRNA* gene, the forward primer 515F (GTGYCAGCMGCCGCGGTAA) and reverse primer 806R (GGACTACNVGGGTWTCTAAT) were used to amplify the V3–V4 regions. Fungi were identified by PCR, using the forward primer ITS5-1737F (GGAAGTAAAAGTCGTAACAAGG) and reverse primer ITS2-2043R (GCTGCGTTCTTCATCGATGC) to amplify the ITS1 (a) region. For each sample, specific barcodes were incorporated into the primers for multiplex sequencing, and the amplification parameters were described previously (Wang X. et al., 2017).

#### 2.3.2 Illumina MiSeq sequencing

Using horizontal gel electrophoresis on PCR products, we recovered amplicons of appropriate sizes from the agarose gels and pooled them in equal amounts to prepare the DNA sequencing library. PCR-free library construction was performed using BioYigene Biotechnology Co., Ltd. (Wuhan, China), and the libraries were sequenced using the MiSeq PE250 (Illumina, San Diego, CA, USA).

#### 2.3.3 Sequencing data analysis

QIIME v.1.9.1^1^ was used to process the amplicon sequencing data. Adapter sequences were trimmed from the raw sequencing data using the Cutadapt plugin. Reads were filtered, denoised, and merged, and chimeras were removed using DADA2 as previously described (Bokulich et al., 2013; Caporaso et al., 2010; Cheng et al., 2023a). Operational taxonomic units (OTUs) were defined based on sequences with 97% similarity, and all sequences were aligned against the Silva library (version SILVA_138.1). Finally, statistical analysis was performed as previously described (Caporaso et al., 2010; de Lipthay et al., 2004; Nilsson et al., 2019), and the linear discriminant analysis effect size (LEfSe) was used to distinguish the groups (Cheng et al., 2024a).

### 2.4 Detection of VOCs by headspace-solid-phase microextraction-gas chromatography–mass spectrometry (HS-SPME-GC–MS)

#### 2.4.1 Headspace–solid-phase microextraction (HS-SPME)

The 500-mg crushed sample, along with 5 mL of saturated NaCl solution and 10 μL of 0.02994 g/L sec-octanol (internal standard material, chromatographically pure; Sigma-Aldrich Chemical Co., St. Louis, MO, USA), was added to a headspace vial (20 mL) following the previously described method, with adjustments (Zhang et al., 2012). VOCs were extracted using a three-phase extraction head (DVB/CAR/PDMS, 50/30 μm) (Supelco, Inc., Bellefonte, PA, USA), with an initial temperature of 50°C, a preheating time of 5 min, an extraction time of 30 min, and a desorption time of 5 min.

#### 2.4.2 Gas chromatography–mass spectrometry (GC–MS) analysis

A gas chromatograph of Agilent 8,890 coupled with a mass spectrometer of Agilent 5,977 equipped with an electron ionization source (Agilent Technologies, Santa Clara, CA, USA) was used for VOC detection. High-purity helium with a flow rate of 0.8 mL/min was used as a carrier gas, a DB-5MS chromatographic column was used (30 m × 0.25 mm × 0.25 mm, Agilent Technologies, Santa Clara, CA, USA), and the shunt ratio was 5:1. Temperature programming was used to operate the equipment as described previously (Fan et al., 2018).

#### 2.4.3 Qualitative and semi-quantitative analyses

Matching and quantification of VOCs were performed based on the National Institute of Standards and Technology database (NIST 05 s). Compounds with a matching degree greater than 80% were selected and combined with the retention time of C7-C40. The relative signal intensity of sec-octanol was used to calculate the percentage area of each peak.

### 2.5 Statistical analysis

SPSS (version 21.0; IBM, Armonk, NY, USA) was used to perform the statistical tests. Pattern recognition was performed using SIMCA-P 14.1 (Umetrics, Umea, Sweden), and principal component analysis (PCA) was performed after preprocessing the data using Pareto scaling (Yan et al., 2013). Orthogonal partial least squares discriminant analysis (OPLS-DA) was employed to identify volatile organic compounds (VOCs) with significant differences between the three replicates of each sample. Significantly different VOCs were identified based on the variable importance in projection (VIP) values, with VIP > 1 and *p* < 0.05. Redundancy analysis (RDA) was performed based on physicochemical parameters, levels of VOCs, and relative abundances of bacterial and fungal communities. Finally, the correlation between the main microbial flora and VOCs was analyzed using Cytoscape 3.5.1 (Yan et al., 2013).

## 3 Results and discussion

### 3.1 Analysis of physicochemical parameters

**Figure 1:**
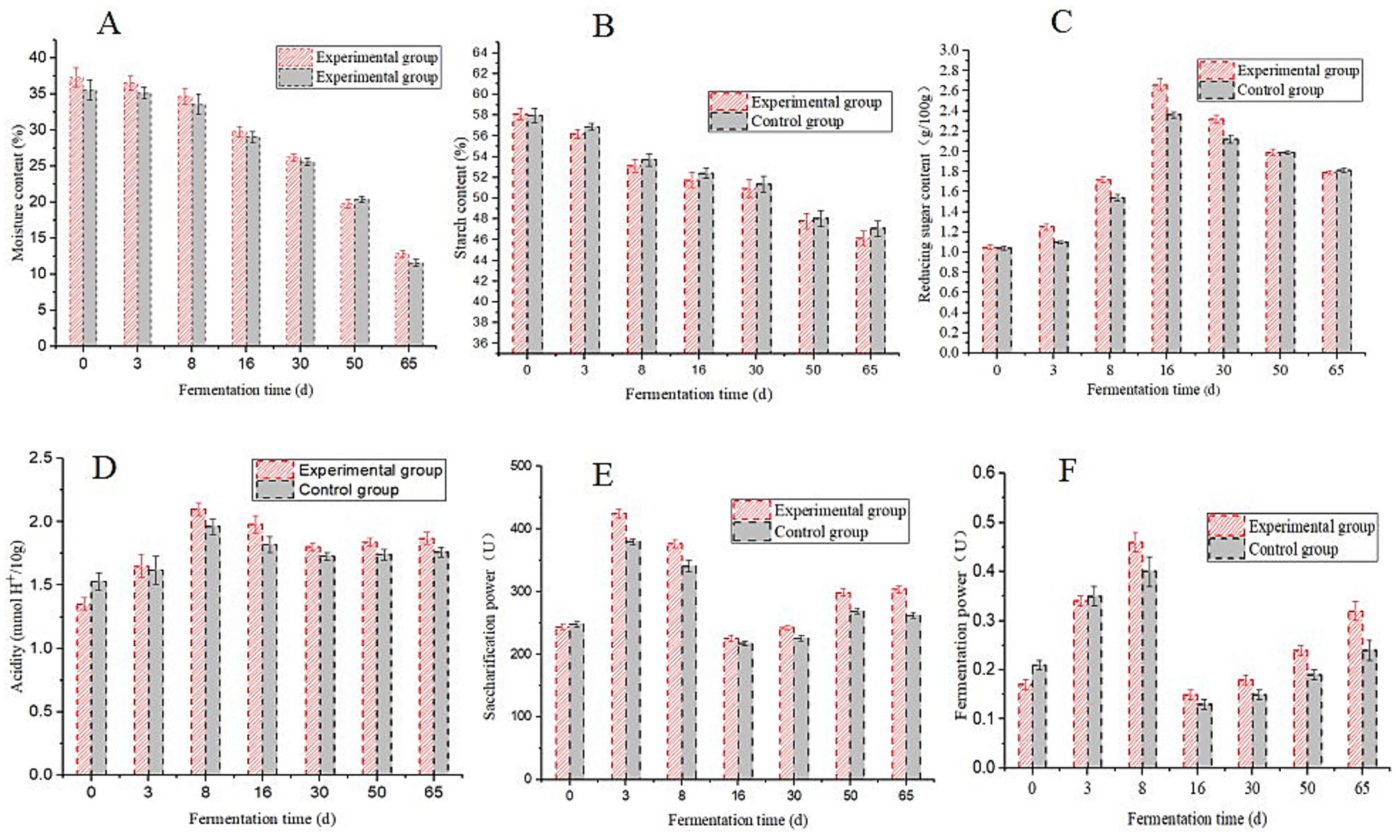
Changes in the main physicochemical parameters during the fermentation process of high-temperature daqu (HTD). **(A)** moisture; **(B)** starch; **(C)** reducing sugar; **(D)** acidity; **(E)** saccharification power; **(F)** fermentation power.

Physicochemical parameters and enzymatic activities are vital indicators of daqu quality (Cheng et al., 2023a; Fan et al., 2018). The moisture content in the HTD began to decrease after 3 d of fermentation (Figure 1A). Variations in moisture content indicate varying degrees of water expulsion from daqu, which may be associated with differences in mycelial spreading after the vaccination of strains (Zhang P. et al., 2024; Zhang Y. et al., 2024). During the HTD fermentation process, the starch content exhibited a gradual decrease (Figure 1B), while the reducing sugar content initially increased and then decreased (Figure 1C). In summary, the starch and reducing sugar contents in the fortified HTD were slightly lower than those in the traditional HTD, indicating that microbial metabolism is relatively active in the fortified HTD.

Acidity increased rapidly during the early fermentation period of the fortified HTD and gradually decreased during the middle and later fermentation periods (Figure 1D). Acidity is regarded as one of the main factors promoting the succession of microbial communities in daqu. Acid-producing microorganisms can produce organic acids in the early period of daqu fermentation, inhibit the growth and reproduction of miscellaneous bacteria, and further affect the assembly of the daqu microecology (Liu et al., 2024), which can reflect the type and content of flavor compounds in daqu (Fan et al., 2018).

In addition, the saccharification power of the fortified HTD was the highest during the early fermentation stage. With an increase in fermentation temperature, the saccharification power began to decline rapidly after 8 d and then increased slowly after the 16th day (Figure 1E). The saccharification power of daqu is affected by temperature and can be improved by filamentous fungi (Cheng et al., 2023a). In the early fermentation period, fungi began to grow and multiply in daqu, undergoing further metabolic processes to produce esterification enzymes. The metabolism of microorganisms was inhibited by an increase in temperature, which resulted in a decrease in saccharification power during the middle fermentation stage. Meanwhile, the fermentation power of fortified HTD reached its highest value (0.46 + 0.02 U) on the 8th day (Figure 1F), which was influenced by the fermentation temperature and the metabolism of yeast (Cheng et al., 2023a). In general, the acidity, saccharification power, and fermentation power of traditional HTD were lower than those of fortified HTD, indicating that the physicochemical parameters of HTD were improved by the application of the *B. halotolerans* strain.

During daqu fermentation, a series of enzymes are produced due to the physiological and biochemical metabolism of microorganisms, including acidic protease enzymes, neutral protein enzymes, liquefying enzymes, saccharifying enzymes, cellulase, pectinase, lipase, tannase, and phytase. *Bacillus* has a powerful hydrolase system, particularly due to its ability to produce amylase. Moreover, *Bacillus*, *Actinomycetes thermophilus*, and *Thermophilic ascomyces* have relatively high protease-producing capacities (Rai et al., 2017; Li et al., 2024). Studies have shown that mold metabolism provides a rich enzyme system for daqu, promotes its fermentation and maturation, and plays a vital role in determining the quality and functional characteristics of daqu (Cheng et al., 2023b). However, research on the relationship between diverse group structures of microorganisms and their enzyme-producing characteristics is limited. Herein, we measured the key enzymes (e.g., amylase and esterase) of daqu and further directly linked microbial functions to physicochemical properties, which will be necessary in future research.

### 3.2 Microbiota in HTD was revealed using sequencing

#### 3.2.1 Sequence statistics, validity, and alpha diversity analyses

The Illumina MiSeq was used to obtain the raw sequence data of the sample microorganisms. After splicing and quality control, 43,669–75,683 effective bacterial sequences were obtained, with an average length of 250–260. After clustering, a total of 5,332 OTU classifications were generated, and the sequencing depth met the species coverage requirements. Meanwhile, 41,063–73,652 effective fungal sequences were obtained, with an average length of 225–250 bp, and 1,621 OTU classifications were generated in total after clustering. The sequencing depth met the species coverage.

**Figure 2:**
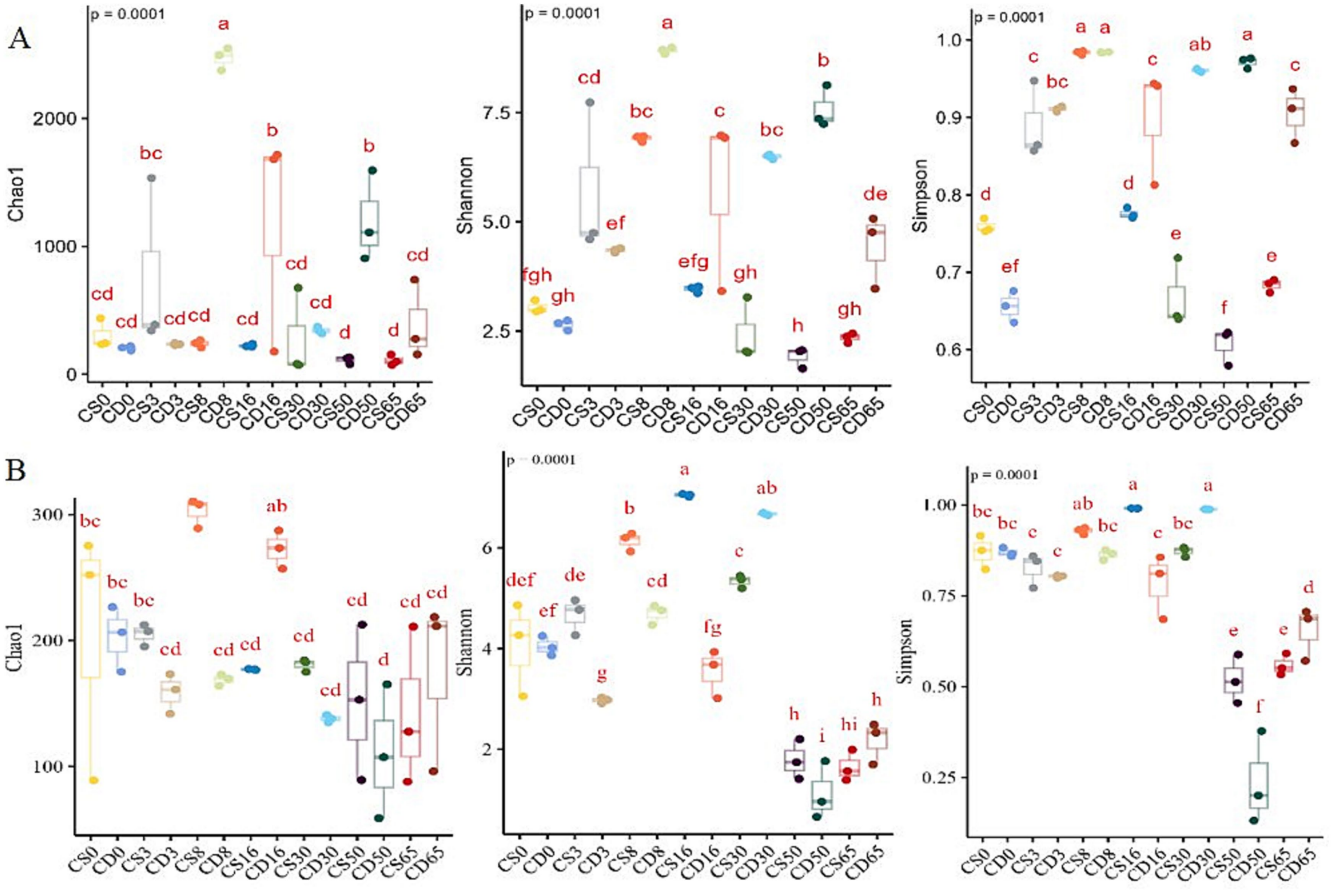
Biodiversity during HTD fermentation is based on changes in the Chao1, Shannon, and Simpson indices. Diversity indices for **(A)** bacteria and **(B)** fungi.

Alpha diversity was used to evaluate the overall diversity of species within habitats, including microbial diversity, represented by the Shannon and Simpson indices, and microbial richness, represented by the Chao1 index (Cheng et al., 2023a). The Chao index at the OTU level was used to calculate coverage, and 99% similarity was used to estimate the total number of species. The *p*-values of the Chao1 index values were all <0.0001 (Figure 2), indicating that the sequencing results of this experiment accurately represented the real situation of the different samples, and the sequencing depth met the species coverage requirements.

Alpha-diversity metrics indicated a decrease in the relative abundance of fungal species during the early fermentation period, followed by an increase in the relative abundance of both bacteria and fungi as the temperature increased during the middle fermentation stage (Figure 2). In addition, the Shannon and Simpson index values revealed a decrease in the uniformity and diversity of the bacterial community in the fortified HTD group over 0–16 days (Figure 2A; Supplementary Table S1) and an increase in the uniformity and diversity of the fungal community in the fortified HTD group as fermentation progressed (Figure 2B; Supplementary Table S2). For both bacterial and fungal communities, uniformity and diversity were high during the middle stage of the fermentation process (8–30 d).

#### 3.2.2 Venn diagram analysis of the OTU distribution

**Figure 3:**
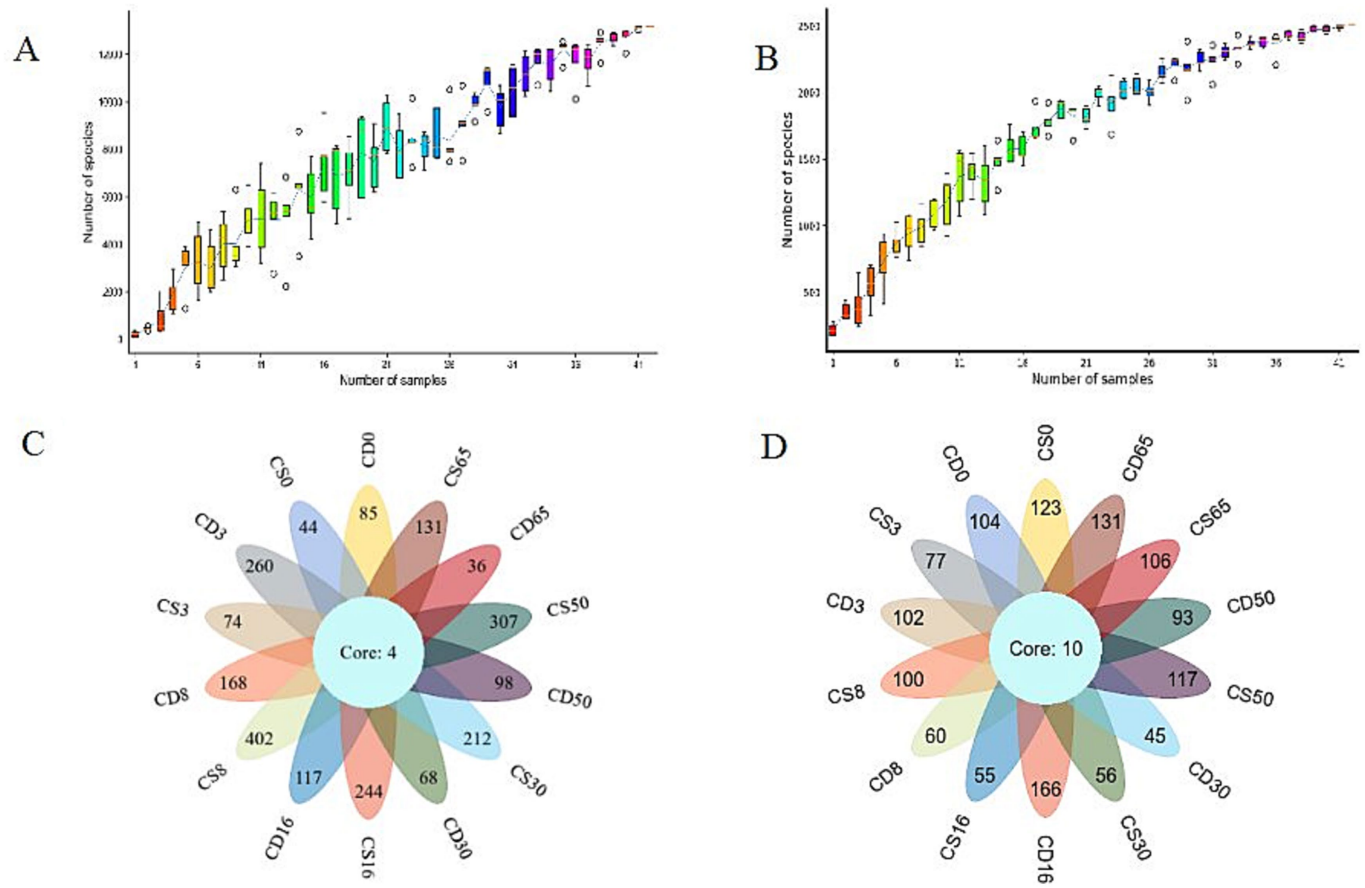
Species accumulation box and Venn diagram of the core operational taxonomic unit distribution of microorganisms in HTD during the culture process. **(A)** Box diagram of bacterial species accumulation; **(B)** box diagram of fungal species accumulation; **(C)** Venn diagram of bacteria; **(D)** Venn diagram of fungi.

The species accumulation box shows that the rate of increase in the number of new species (OTU number) was observed as the sample size continuously expanded throughout sampling. As the number of samples increased, the observed rate of increase in the number of new species (OTU number) leveled off (Figures 3A,B), indicating that the sequencing data were representative.

The same OTUs were classified based on sequence similarities greater than or equal to 97%, and the number of species and overlap between different samples were analyzed using classification statistics at the OTU level during HTD fermentation. Four bacterial OTUs overlapped in different samples (Figure 3C), accounting for 0.18% of the total bacterial OTUs. A total of 402 OTUs were specific to CS8, accounting for 17.89% of the total number of bacterial OTUs, indicating that the bacterial diversity of CS8 was the highest among the samples. Additionally, 10 fungal OTUs overlapped between the different samples (Figure 3D), accounting for 0.75% of the total number of fungal OTUs identified. For CD16, the maximum number of sample-specific OTUs was 166, accounting for 12.43% of the total number of fungal OTUs, indicating that the fungal diversity of CD16 was the highest among the samples.

For samples of fortified HTD, CS8 had the highest number of bacterial OTUs (402), and CS50 had the highest number of fungal OTUs (117), indicating that bacterial diversity was greatest in CS8 and fungal diversity was greatest in CS50. It has been reported that the native microbial community structure in the MTD is affected by inoculation with *Bacillus* strains and the abundance of 22 microbial genera changes (Xu B. et al., 2022). In general, fortification with *B. halotolerans* had a significant impact on bacterial diversity but had minimal effect on fungal diversity in HTD during fermentation.

#### 3.2.3 Analysis of microbial community based on the phylum and genus levels

**Figure 4:**
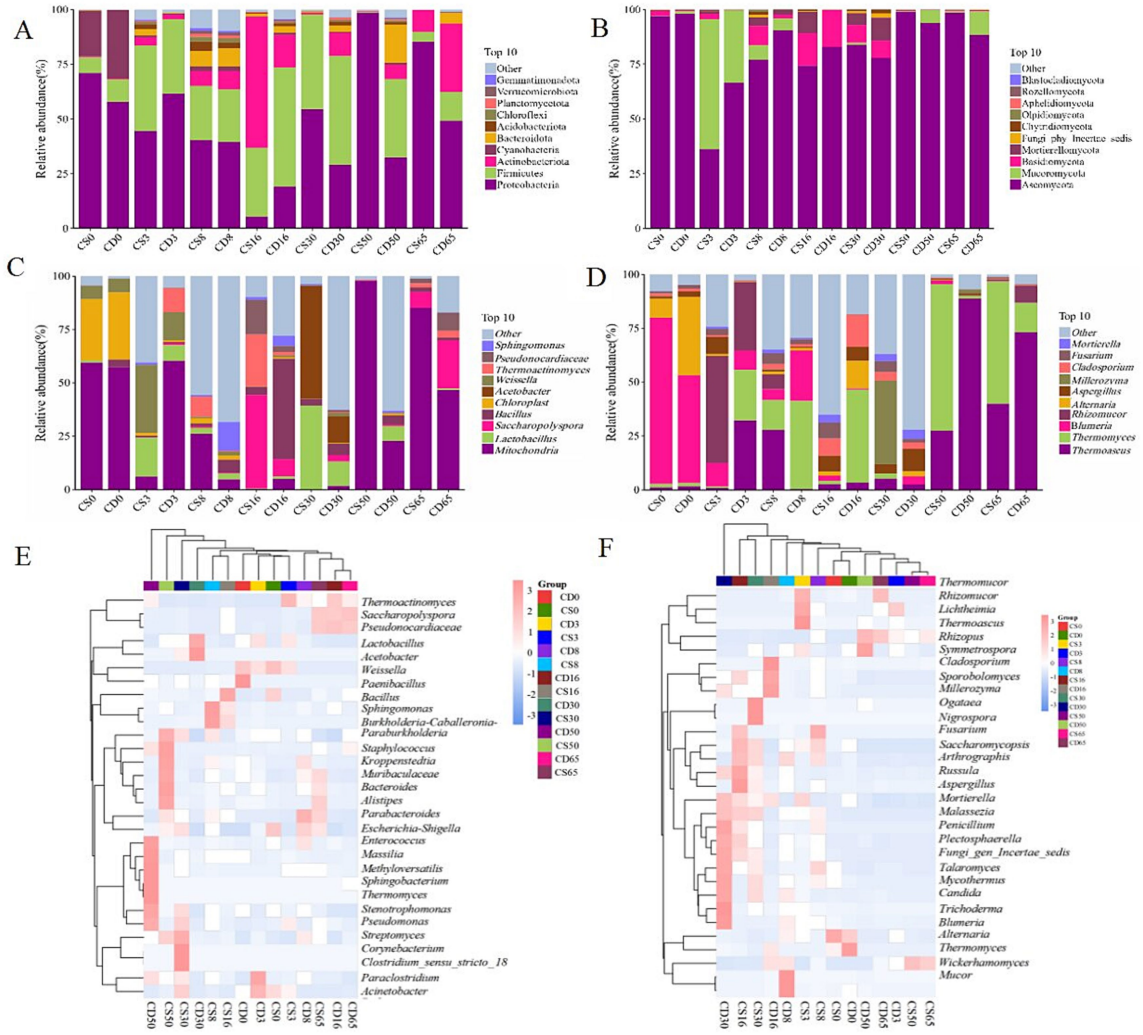
Microbial community structure in HTD during fermentation at the phylum and genus levels. **(A)** Bacterial phyla; **(B)** fungal phyla; **(C)** bacterial genera; **(D)** fungal genera; **(E)** heatmap of bacterial genera; **(F)** heatmap of fungal genera.

Community structure was measured at different taxonomic levels. The bacterial community consisted of 22 phyla, 714 genera, and 134 species, while the fungal community was classified into 9 phyla, 125 genera, and 148 species. The relative abundances of Proteobacteria and Firmicutes, regarded as the dominant bacterial phyla, were >1.00% (Figure 4A). In addition, the relative abundance of Firmicutes was >50.00%, making it the predominant bacterial phylum (Figure 4A). As shown in Figure 4B, the relative abundances of Ascomycota and Basidiomycota were >1.00%, indicating them as the dominant fungal phyla. In addition, the relative abundance of Ascomycota was >36.00%, making it the absolute dominant phylum. Collectively, two dominant phyla were observed in HTD during different fermentation periods, and differences in the relative abundance of bacterial and fungal phyla were noted in samples from different groups.

Microorganisms in HTD at different stages of fermentation can be classified into the main bacterial genera: *Weissella*, *Bacillus*, and *Lactobacillus*. *Lactobacillus* was regarded as the dominant strain, with a relative abundance >1.00% (Figure 4C). For the fortified HTD samples, the relative abundance of *Lactobacillus* was 39.34% (CS30) during the middle fermentation period, whereas in traditional HTD, it was 11.47% (CD30). The relative abundance of *Weissella* was 31.86% (CS3) at the beginning of the fermentation period, whereas in traditional HTD, it was 13.2% (CD3). This shift may be driven by the application of *B. halotolerans*, which likely influenced the evolution of the community structure. During fermentation, microorganisms continuously produce saccharolytic enzymes that degrade starch into reducing sugars, which are further converted into lactic acid by *Weissella* via the pyruvate metabolic pathway (Tang and Peng, 2024). *Lactobacillus* species can metabolize and produce lactic acid during baijiu fermentation, which plays a vital role in promoting the production of ethyl caproate aroma (Cheng et al., 2023b; Xu Y. et al., 2022). In comparison, the relative abundance of *Bacillus* initially increased, then decreased, and finally stabilized during HTD, which is consistent with existing research results (Gong et al., 2025). *Bacillus* is a primary component of microbial communities in fermented grains (Cheng et al., 2024a). As the primary functional bacterial genus, *Bacillus* has a significant impact on the microbial community structure and flavor characteristics of grains during baijiu fermentation (Tong et al., 2022, 2024).

The microorganisms at different times during fermentation included four main fungal genera: *Blumeria*, *Alternaria*, *Thermoascus,* and *Aspergillus* (Figure 4D). The relative abundance of *Blumeria* was highest at the beginning of fermentation (77.13% in CD0 and 49.92% in CS0) and gradually decreased with increasing fermentation time. Meanwhile, the relative abundance of *Blumeria* was the lowest at the end of fermentation (0.01% in CD65 and 0.23% in CS65). In contrast, the relative abundance of *Thermoascus* first increased and then decreased during HTD fermentation, indicating a positive correlation between temperature and *Thermoascus*. It reported that *Thermoascus* is a heat-tolerant fungus that can grow at high temperatures, which makes it particularly active during HTD fermentation. *Thermoascus* can produce amylases that break down starch into fermentable sugars, creating a favorable environment for other microorganisms and promoting ethanol production (Gong et al., 2025).

As shown in Figure 4E, at least two bacterial genera had relative abundances >1.00% during HTD fermentation. For CD65, the relative abundances of *Saccharopolyspora* and *Pseudonocardiaceae* were >3.00%, and the relative abundances of *Bacillus* were 2.18% (CS65) and 1.36% (CD65), indicating that fortification with *B. halotolerans* increased the relative abundance of *Bacillus*. As shown in Figure 4F, the relative abundances of the three fungal genera were more than 1.00%, observed during HTD fermentation. At the end of fermentation, the relative abundances of *Thermoascus*, *Rhizomucor*, *Thermomyces*, and *Thermomucor* were all >2.00%. In the CS group (experimental group), the relative abundance of *Blumeria* significantly reduced from 77.13 to 0.23%, which was lower than that in the CD group (control group), which showed a significant reduction from 49.92 to 0.01%. Thus, the relative abundances of the Firmicutes phylum, the *Bacillus* genus, the Ascomycota phylum, and the *Blumeria* genus differed significantly, with Firmicutes and Ascomycota being the dominant phyla. The results of this study indicate that the application of *B. halotolerans* regulates the microbial community structure of the daqu ecosystem.

The abundance of *Rhizopus*, *Bacillus*, *Aspergillus*, and *Rasamsonia*, which secrete glycosylase, increased after fortification with *B. licheniformis* and *B. velezensis* (Xu B. et al., 2022). Within microbial cells, pyruvic acid produced from glucose is converted into lactic acid by many lactic acid bacteria. *Leuconostoc* and *Kroppenstedtia* produce acetolactate synthase, which catalyzes the fermentation of the remaining pyruvic acid, thereby entering the fermentation process related to pyruvate metabolism and the TCA cycle. Additionally, the abundance of *Leuconostoc* and *Kroppenstedtia* decreased after inoculation with *Bacillus* in the daqu, and the reductase enzyme secreted by *Aspergillus* considerably increased the concentration of aromatic compounds in the fortified HTD, as represented by phenylethanol (Xu B. et al., 2022).

#### 3.2.4 LEfSe analysis of microbial community at the species level and association diagram of differential genera of microbial

**Figure 5:**
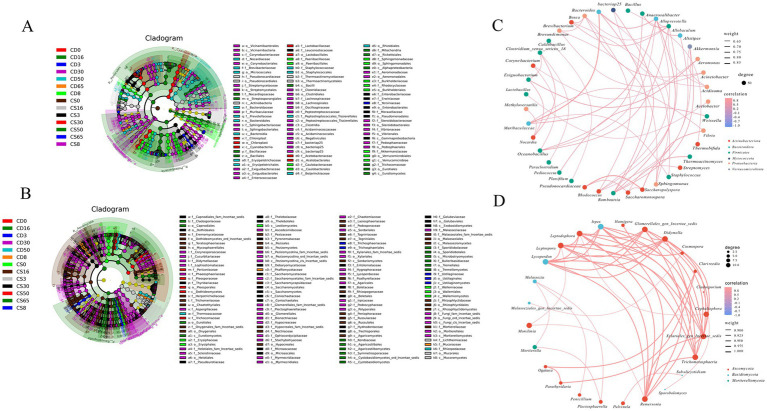
LEfSe analysis of the microbial community at the species level and association diagram of differential genera of microbial. **(A)** LEfSe analysis of bacteria species; **(B)** LEfSe analysis of fungi species; **(C)** association diagram of bacteria genera; **(D)** association diagram of fungi genera.

LEfSe analysis was used to understand the differences in microflora and microbial species among the groups (Cheng et al., 2024a). There were 305 bacterial species, including o__Vicinamibacterales, c__Vicinamibacteria, and f__Nocardiaceae (Figure 5A), that were upregulated. A total of 524 fungal species were upregulated, including f__Cladosporiaceae, o__Capnodiales, and o__Dothideales (Figure 5B). There were significant differences between the bacterial species *Bacillus coagulans*, *Bacillus thermoamylovorans*, and *Bacillus thermolactis* (Figure 5A) and the fungal species *Blumeria graminis*, *Arthrographis kalrae*, and *Aspergillus teporis* (Figure 5B).

For the bacterial communities, the differential bacterial phyla included Actinobacteria, Bacteroidota, Firmicutes, Myxococcota, Proteobacteria, and Verrucomicrobiota. The 39 differential bacterial genera were divided into two parts in the correlation network (Figure 5C). For example, *Bacteroides* was positively correlated with *Muribaculaceae,* and *Pseudonocardiaceae* was positively correlated with *Saccharopolyspora.* The other genera formed the second group, and most of the microbes in the second group were weakly correlated. Based on the correlation network, the differential fungal phyla included Ascomycota, Basidiomycota, and Mortierellomycota, and the 25 fungal genera were divided into two groups (Figure 3D). For example, *Remersonia*, *Trichometasphaeria*, *Xylariales_gen_Incertae_sedis*, *Cephaliophora*, *Didymella*, *Glomerellales_gen_Incertae_sedis*, and *Monilinia* were strongly positively correlated. The other genera formed the second part, consisting of *Hamigera*, *Cosmospora*, *Clarireedia*, *Cladosporium*, *Subulicystidium*, *Sporobolomyces,* and *Malasseziales_gen_Incertae_sedis*, which showed relatively weak positive correlations.

### 3.3 Analysis of changes in VOCs in HTD during fermentation

Alcohols, acids, and esters are regarded as the main flavor compounds in baijiu, and their types and concentrations vary widely depending on the aroma type and manufacturer and are also related to the saccharifying agent (including daqu, xiaoqu, and fuqu) (Cheng et al., 2022; Zhang J. et al., 2022). Eighty-two VOCs were detected in HTD, and they were clustered into five groups based on differences in their chemical structures. These included 8 alcohols, 7 acids, 14 esters, 5 aldehydes and ketones, 31 aromatic and phenolic compounds, and 17 other compounds (Supplementary Table S3). Esters, aromatic compounds, and phenolic compounds are the main flavor compounds in HTD. It has been reported that an inverse relationship exists between the ester content and the peak temperature of different types of daqu, such as ethyl acetate, phenethyl acetate, ethyl valerate, and pentyl acetate, which have special floral and fruity aromas (Deng et al., 2023). Overall, the unique main flavor substances of the fortified HTD (CS group) included butanediol, phenylethyl acetate, nonanal, and methyl benzoate. In contrast, the unique main flavor substances of the traditional HTD (CD group) included furfural, 3-phenylfuran, and trimethylpyrazine (Supplementary Table S3).

**Figure 6:**
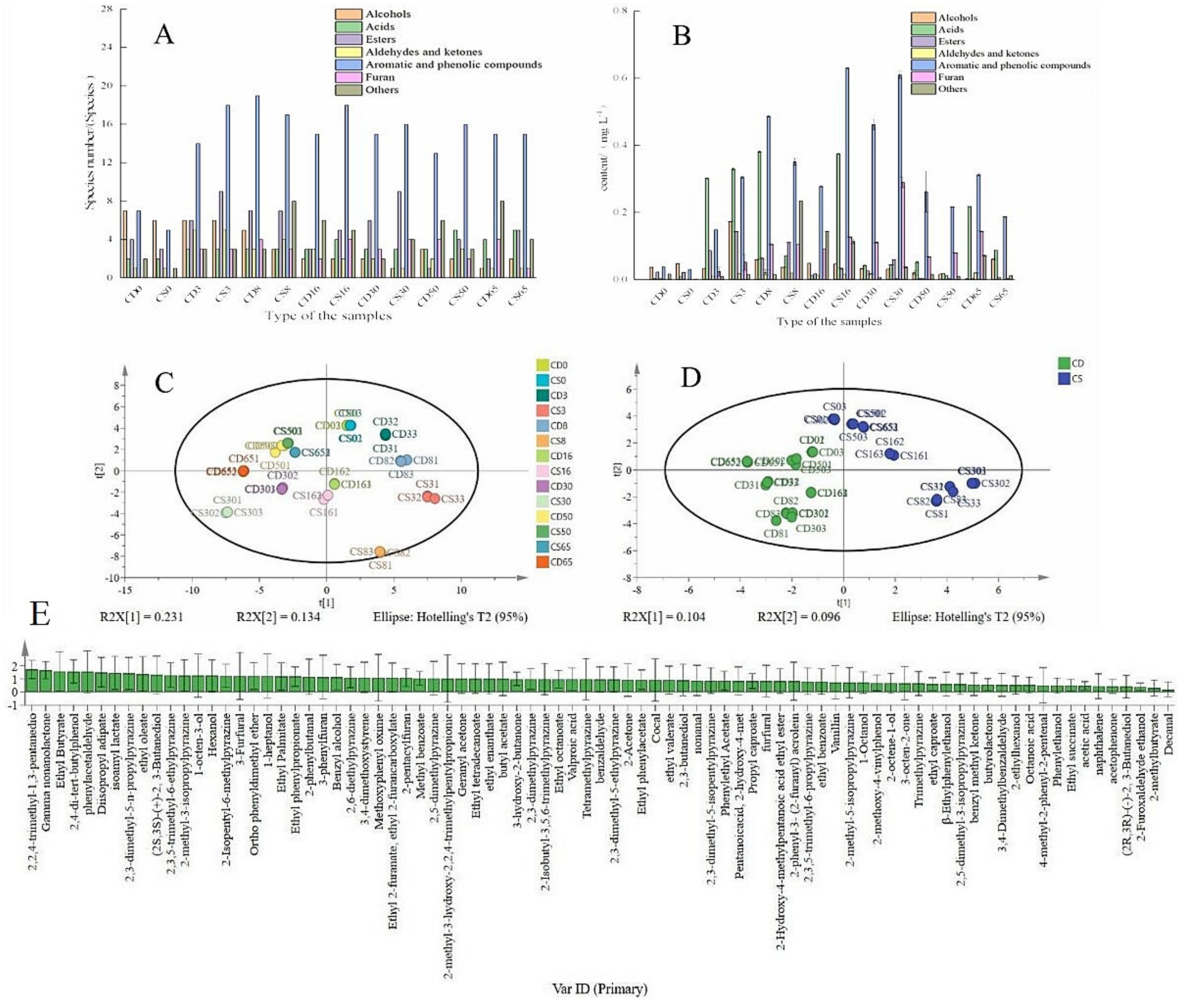
Categories and contents of VOCs in fermented grains. **(A)** Quantity of VOCs; **(B)** content of VOCs; **(C)** PCA; **(D)** OPLS-DA; **(E)** VIP.

The number of aromatic and phenolic compounds was highest among the detected VOCs, and the number of esters increased during fermentation (Figure 6A). During the middle fermentation period, the contents of most VOCs, including acids, alcohols, and aromatic and phenolic compounds, increased (Figure 6B). Alcohols and ester compounds are important flavor substances that account for a high proportion of all organic substances in baijiu (Xu Y. et al., 2022). With the inoculation of *B. halotolerans*, the effects of microbial interactions are reflected in changes in the types and contents of enzymes and flavor substances related to microbial metabolism (Fan et al., 2018). The alcohol and ester contents were higher in fortified HTD than in traditional HTD, and the majority of the volatiles in daqu exhibited low sensory thresholds, which, combined with other low-abundance volatiles, may contribute to its distinctive aroma. Furthermore, these micro-volatiles are the main contributors to the flavor of baijiu (Ren et al., 2024; Lan et al., 2024).

However, the acid, aromatic, and phenolic compounds, aldehyde, and ketone contents were lower in fortified HTD than in the traditional HTD (Figure 6B) at the end of fermentation. In summary, the flavor substances in HTD changed during different periods of fermentation with the application of *B. halotolerans*, which may be related to alterations in physicochemical parameters and microbial communities (Fan et al., 2018; He et al., 2019a, 2019b). It is worth noting that several types of pyrazine compounds were detected, including trimethyl pyrazine, tetramethyl pyrazine, and pyrazine compounds with special flavor substances and pharmacological effects, which impart a nutty and baked aroma to baijiu (Cheng et al., 2024b).

As shown in Figure 6C, within the first two principal components, the cumulative contribution rate of the corresponding features reached 36.5%, indicating that a substantial portion of the flavor-related compound information was encapsulated. As shown in Figure 6D, for the first two principal components, the cumulative contribution rate of the corresponding features increased to 20.0%, confirming the capability of the model to accurately reflect a significant 20.0% alteration in the dataset. In addition, all samples were within the 95% confidence interval of Hotelling’s T2, confirming the stability and dependability of the model. As shown in Figure 6D, the samples clustered within specific regions when OPLS-DA was applied, indicating discernible dissimilarities among the VOCs in the samples. The VIP values were regarded as the weight values ascribed to the variables within the OPLS-DA model, and VIP > 1 indicated the pivotal differentiating components in different samples (Cheng et al., 2024b). Thirty-six compounds with VIP > 1 were identified, including ethyl butyrate (VIP = 1.57), ethyl oleate (VIP = 1.38), and benzyl alcohol (VIP = 1.13), indicating that they were distinctive compounds that accentuated disparities among samples (Figure 6E). Overall, bioturbation of fortified daqu is a feasible approach for enhancing flavor metabolism through interspecies interactions among functional microbiota during the fermentation of daqu, which is of great importance for regulating baijiu fermentation by bioturbation.

### 3.4 RDA of the dominant microbial community with physicochemical properties and VOCs

**Figure 7:**
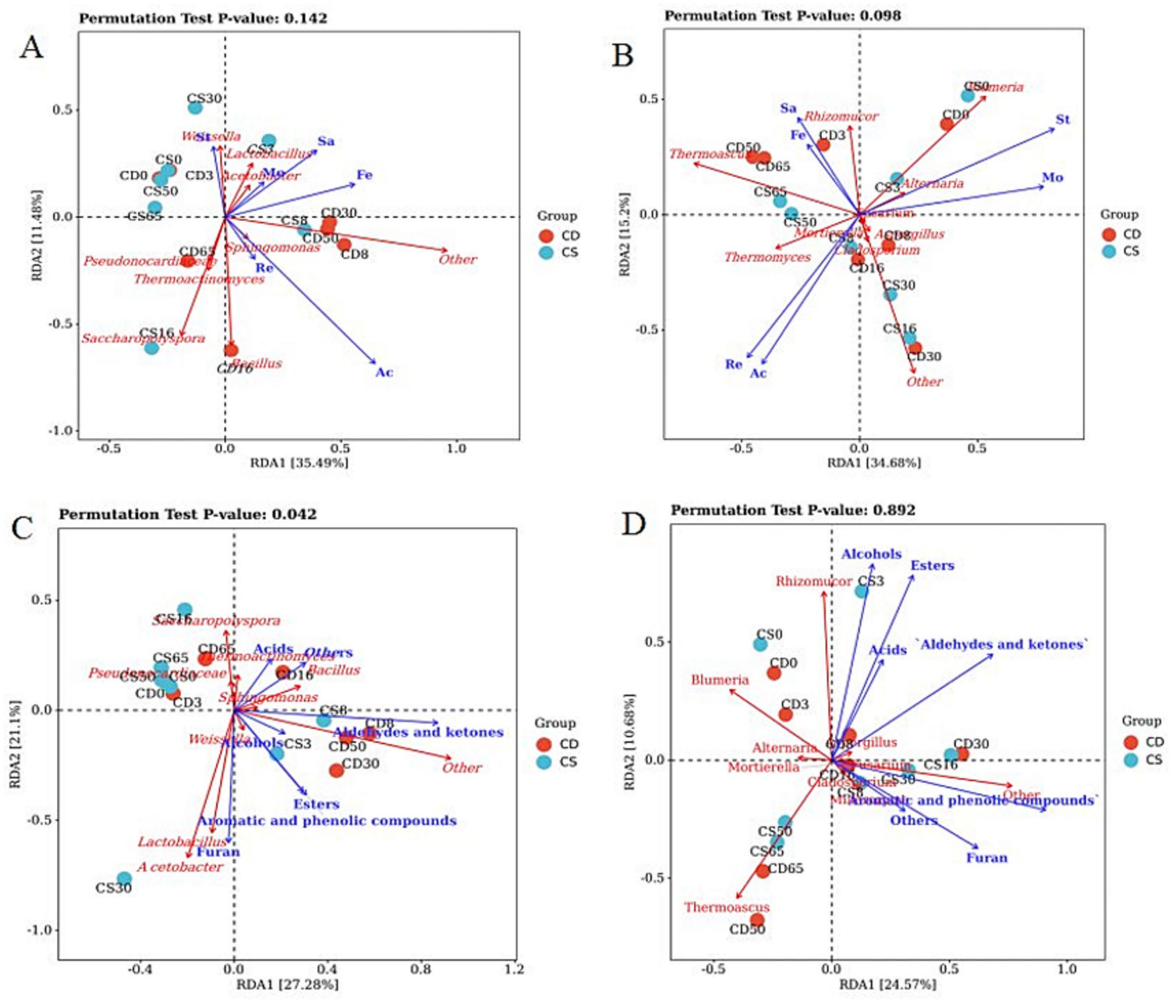
Redundancy analysis (RDA) of the dominant microorganisms with physicochemical parameters and VOCs. **(A)** RDA of physicochemical parameters and bacterial genera; **(B)** RDA of physicochemical parameters and fungal genera; **(C)** RDA of VOCs and bacterial genera; and **(D)** RDA of VOCs and fungal genera. Mo, moisture; St: starch; Re: reducing sugar; Ac: acidity; Sa, saccharification power; Fe: fermentation power.

RDA was used to analyze the physicochemical properties, VOCs, and relative abundances of the dominant microbial community. The top two RDA axes could explain a total of 46.97 and 49.88% of the variation in metabolites, respectively, indicating partial correlations between the physicochemical parameters and microbial communities (Figures 7A,B). The influence of acidity, saccharifying power, and fermenting power on the composition and function of the microbiota was greater than that of other factors. Based on the *p*-values of the permutation test, physicochemical parameters had a greater effect on the composition and function of the bacterial flora (*p* = 0.142) than on those of the fungal flora (*p* = 0.098). The relative abundance of *Bacillus* positively correlated with acidity and reduced sugar content. Additionally, the abundances of *Rhizopus* and *Thermoascus* were positively correlated with saccharification and fermentation power (Figures 7A,B; Supplementary Table S4). Microorganisms of filamentous fungi contribute to the saccharification power of daqu (Cheng et al., 2023a; Fan et al., 2020), *Rhizomucor* contributes to the liquefying activity and saccharifying power of daqu (Cheng et al., 2023a; Fan et al., 2020), and *Thermomyces* has been reported to be a high producer of thermophilic enzymes for carbohydrate degradation (He et al., 2019a).

The top two RDA axes explained 48.38 and 35.25% of the variation in metabolites in the groups, respectively, indicating partial correlations between the microbial communities and VOCs (Figures 7C,D). The effects of fungal flora (*p* = 0.892) on VOCs were greater than those of bacterial flora (*p* = 0.042) based on the *p*-values of the permutation test. Such alcohols, acids, and esters, at the levels of most VOCs, were positively correlated with the main genera in the HTD, including the bacterial genera *Lactobacillus*, *Acetobacter*, and *Bacillus,* as well as the fungal genera *Rhizomucor*, *Thermoascus,* and *Blumeria* (Figures 7C,D; Supplementary Table S4). RDA results also showed that *Lactobacillus* was highly positively correlated with these esters, and these esters play a vital role in contributing fruity and floral aromas to baijiu (Cheng et al., 2023b). As core functional microorganisms, *Lactobacillus* species are responsible for increasing acidity, as they can metabolize acetic acid, lactic acid, and ethanol through heterolactic fermentation (Cheng et al., 2023c; Xu S. et al., 2022). Daqu is enriched in lactic acid bacteria (such as *Lactobacillus*) for fermentation and the development of flavor compounds. In contrast, lactic acid bacteria play important roles in the production of various metabolites and the baijiu flavor (Ali et al., 2024). Conversely, *B. subtilis* and *B. licheniensis* can metabolize various flavor compounds that are important for the quality of the Maotaiflavor in baijiu, such as various acids, pyrazines, and methyl esters (Cheng et al., 2024b; Li et al., 2014; Zhang et al., 2007). Most previous studies have focused on the influence of bacterial communities on VOCs. In the present study, aside from the bacterial genera *Lactobacillus*, *Acetobacter,* and *Bacillus*, the levels of most VOCs were also positively correlated with the fungal genera *Rhizomucor*, *Thermoascus*, and *Blumeria*.

Overall, the fortified application of *B. halotolerans* had varying degrees of influence on the microbial community structure and volatile aroma substances of HTD. It has been reported that bacteria and yeast are typically cultured as liquid strains in specialized culturing equipment and then introduced into cooked and cooled raw materials at a mass ratio of 5–10% (Cheng et al., 2022). Optimizing the vaccination volume and ratio is crucial for the quality of fortified HTD. In the present study, as described in the fortified HTD manufacturing section, the use of single-concentration inoculation, a lack of dose-effect verification, and the absence of biological replicates may affect statistical reliability (as seen in the RDA result with *p* = 0.892). Therefore, dose-effect verification and biological replicates are necessary for future research. In addition, the application of distance-based RDA (db-RDA) and Mantel tests to validate correlations between microbial communities and VOCs and incorporating additional environmental factors (e.g., temperature and pH) to increase explanatory power will be necessary in future research. This aims to further support the robustness and transferability of the results.

### 3.5 The statistics and genera composition of metabolic pathways

**Figure 8:**
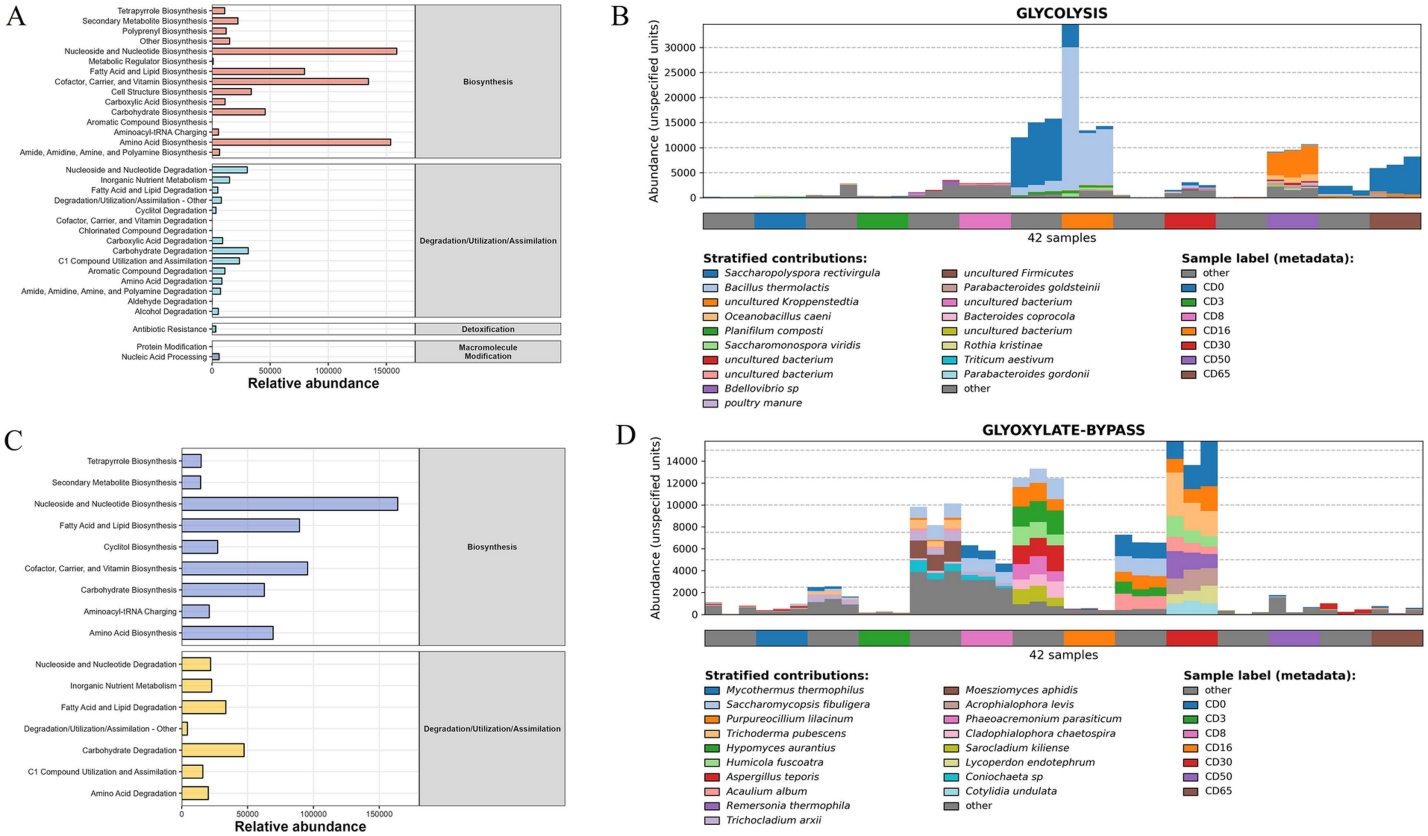
The statistics and genera composition in metabolic pathways. **(A)** Statistics of bacterial metabolic pathways; **(B)** bacterial genera composition in metabolic pathways of glycolysis; **(C)** statistics of fungal metabolic pathways; **(D)** fungal genera composition in metabolic pathways of glyoxylate-bypass.

The statistics and species compositions of the metabolic pathways are shown in Figure 8. For the bacterial genera, 33 metabolic pathways (Figure 8A) were classified into biosynthesis, degradation/utilization/assimilation, detoxification, and macromolecule modification categories. The metabolic pathways with relatively high abundance were amino acid biosynthesis (153675.19), cofactor, carrier, and vitamin biosynthesis (134550.94), and nucleoside and nucleotide biosynthesis (158877.79). For the critical metabolic pathways of glycolysis (Figure 8B), the bacterial genera that contributed a relatively high abundance to this metabolic pathway included *Saccharopolyspora rectivirgula*, *Bacillus thermolactis*, *uncultured kroppenstedtia*, and *Oceanobacillus caeni*.

For the fungal genera, 16 metabolic pathways (Figure 8C) were classified into biosynthesis, degradation, utilization, and assimilation. The metabolic pathways with relatively high abundance were nucleoside and nucleotide biosynthesis (163979.17), cofactor, carrier, and vitamin biosynthesis (95611.56), and fatty acid and lipid biosynthesis (89417.74). For the critical metabolic pathways of the glyoxylate-bypass (Figure 8D), fungal genera that contribute to a relatively high abundance of this metabolic pathway include *Mycothermus thermophilus*, *Saccharomycopsis fibuligera*, and *Purpureocillium lilacinum*.

In this study, we investigated the changes in the physicochemical properties, microbial abundance, and VOCs in HTD after the fortified application of *B. halotolerans*. Furthermore, the statistics and species composition of the metabolic pathways were analyzed. However, specific microbial functions and the role of related enzymes in metabolic pathways require further transcriptomic and proteomic analyses. Further studies are needed to understand the interaction between *B. halotolerans* and other microorganisms in HTD after the fortified application of *B. halotolerans*, as well as the impact of these interactions on the changes in physicochemical properties and VOCs in HTD during fortification.

## 4 Conclusion

Microbes and their metabolic activities play a critical role in determining HTD quality. In this study, we analyzed the physicochemical properties, microbial communities, and VOCs of HTD to evaluate the effects of fortification with *B. halotolerans*. The results revealed that the acidity, saccharification power, and fermentation power of the fortified HTD were higher than those of traditional HTD. In general, fortification with *B. halotolerans* was favorable for increasing the relative abundance of Firmicutes and *Bacillus* and significantly altered the relative abundance of Ascomycota and *Blumeria*. For the fortified HTD, the content of certain VOCs, including esters and alcohols, increased at the end of the fermentation process. Physicochemical properties influenced bacterial flora more than fungal flora, and the effects of fungal flora on VOCs were greater than their effects on bacterial flora. In summary, fortification with *B. halotolerans* controlled microbial metabolism by altering the relative abundance of certain microorganisms and enhancing the production of certain VOCs, which were also affected by the physicochemical properties of HTD. These results will help promote and guide the strategic application of functional strains in daqu fermentation. Further insights into the function of the microbial community in HTD are required to better understand the relationship among physicochemical properties, microbes, and flavor compounds, which are critical for improving HTD quality and controlling the brewing process.

## Data Availability

The datasets presented in this study can be found in online repositories. The names of the repository/repositories and accession number(s) can be found in the article/Supplementary material.

## Footnotes

^1^ http://qiime.org/scripts/split_libraries_fastq.html
