# Supplementary material for: Effects of the improved application of Bacillus halotolerans on the microbial community and volatile components of high-temperature daqu
Source: Front Microbiol. 2025 Jun 27;16:1626160. doi: 10.3389/fmicb.2025.1626160 (PMC12245780; doi:10.3389/fmicb.2025.1626160)
Supplement: Supplementary file 1 [file Table_1.docx]

**Table S1.** Alpha diversity indices of bacterial metagenomes of samples.

| **Sample** | **Chao1** | **Faith_pd** | **Goods_coverage** | **Observed_species** | **Pielou_e** | **Shannon** | **Simpson** |
| --- | --- | --- | --- | --- | --- | --- | --- |
| CS0-1 | 438.20917481382713 | 50.78717627033348 | 0.9988892754199383 | 423.2 | 0.3682227033801424 | 3.212787214049689 | 0.7694936455408511 |
| CS0-2 | 243.091029870266 | 33.649220574454276 | 0.9995452738296391 | 237.4 | 0.3781970800704018 | 2.984394034044466 | 0.7550960503121595 |
| CS0-3 | 235.48250565205404 | 24.409863414993755 | 0.9996123645760859 | 231.0 | 0.3755176055318356 | 2.9484600024450733 | 0.7527099752142089 |
| CD0-1 | 219.87443851971824 | 30.285716258685078 | 0.9995427889871781 | 211.2 | 0.35536479109390445 | 2.7442519241495593 | 0.6758792874745239 |
| CD0-2 | 187.3212561947856 | 20.97113415955176 | 0.9996993340622204 | 182.8 | 0.33446394556957915 | 2.51316706957509 | 0.6349785214870731 |
| CD0-3 | 208.10681951089845 | 31.778975011708326 | 0.9996744856376105 | 202.9 | 0.3504267837934191 | 2.6858675931123677 | 0.656292823892534 |
| CS3-1 | 342.1537445887446 | 41.70397423988934 | 0.9999105456714042 | 341.3 | 0.5472450317610846 | 4.6050105858018675 | 0.8569526678682362 |
| CS3-2 | 387.4631349206349 | 46.29156826040047 | 0.9998856972467947 | 385.8 | 0.552943779316797 | 4.750729652493741 | 0.8643499953434828 |
| CS3-3 | 1534.491020580297 | 98.77595185693104 | 0.9997838187058941 | 1532.3 | 0.730948255029748 | 7.734516629104296 | 0.947473429177732 |
| CD3-1 | 228.50992825570628 | 26.053434663287447 | 0.9994806679256534 | 222.1 | 0.5600274041969225 | 4.365425903701532 | 0.9108164177248532 |
| CD3-2 | 235.52306664881206 | 29.472081814080504 | 0.9994260013915118 | 227.9 | 0.5615632779947064 | 4.398268943351501 | 0.9144413578216511 |
| CD3-3 | 243.869660717175 | 29.361383128499263 | 0.9993539409601432 | 235.8 | 0.5450901803470394 | 4.296031861200253 | 0.9074134128847129 |
| CS8-1 | 208.0 | 32.96412200453328 | 1.0 | 208.0 | 0.9037880970008592 | 6.959565758928569 | 0.9857277574295061 |
| CS8-2 | 267.2185714285714 | 36.7070981845613 | 0.9999428486233972 | 266.8 | 0.8475528683066493 | 6.830946635913776 | 0.9808533966312106 |
| CS8-3 | 243.9163492063492 | 30.428204351599998 | 0.9999478183083192 | 243.6 | 0.8764266285446418 | 6.948632243729344 | 0.9846492299715003 |
| CD8-1 | 2548.4810713683883 | 173.73701939556253 | 0.9979798230792168 | 2530.4 | 0.795163417747236 | 8.989439219938593 | 0.9846483739468539 |
| CD8-2 | 2375.394581917453 | 171.9713048958218 | 0.9982208527979326 | 2360.0 | 0.7895648413575488 | 8.846732763690445 | 0.9836821755199061 |
| CD8-3 | 2494.0437025285046 | 173.534087040266 | 0.9977412782029619 | 2471.4 | 0.7928955553768343 | 8.936814061491432 | 0.9841668256297357 |
| CS16-1 | 219.1 | 33.30935071063828 | 0.999990060630156 | 219.0 | 0.4493784650999101 | 3.4938218753222188 | 0.783453611382257 |
| CS16-2 | 239.11666666666665 | 34.803164608366686 | 0.999962727363085 | 238.7 | 0.446482514920551 | 3.526788788020089 | 0.7731705175114042 |
| CS16-3 | 214.9 | 35.92074864128908 | 0.9999726667329292 | 214.7 | 0.4352235722798377 | 3.37131796160721 | 0.7702598894778824 |
| CD16-1 | 177.78843158451852 | 26.52718228442392 | 0.9995701222542491 | 172.0 | 0.46026507570645514 | 3.4180120282535924 | 0.8126843994650226 |
| CD16-2 | 1680.485924872689 | 135.00451910562214 | 0.9983997614551239 | 1665.9 | 0.6520967828080759 | 6.9787936099519765 | 0.9438320613603999 |
| CD16-3 | 1716.374786986058 | 135.68424287969125 | 0.9981587317364079 | 1698.5 | 0.6454896331684624 | 6.926127897003985 | 0.9406315992127972 |
| CS30-1 | 675.5527206427771 | 75.55669563602501 | 0.9982978829142232 | 654.6 | 0.3501359572551208 | 3.2753255331235254 | 0.7183888749752639 |
| CS30-2 | 72.84313852813852 | 19.60574543626536 | 0.9997465460689791 | 68.8 | 0.3306085353194582 | 2.018078436912836 | 0.6394946075683189 |
| CS30-3 | 81.04166051698559 | 17.41509494607598 | 0.9996993340622204 | 76.4 | 0.32596206727517063 | 2.0389370736855947 | 0.6434641199368674 |
| CD30-1 | 319.15 | 45.161177945382356 | 0.9999850909452339 | 319.0 | 0.7735510062313831 | 6.433942896619411 | 0.9588565391703 |
| CD30-2 | 339.8833333333333 | 41.48286053440832 | 0.9999478183083191 | 338.8 | 0.7782713018575509 | 6.540816921969106 | 0.9608592936679339 |
| CD30-3 | 372.07000000000005 | 50.604402317198186 | 0.9999254547261703 | 369.4 | 0.7635904445260848 | 6.512692932124376 | 0.9628633157785218 |
| CS50-1 | 79.85038461538463 | 12.10778785264058 | 0.9997415763840574 | 76.3 | 0.2631510472174873 | 1.6454042788038752 | 0.5791241000219701 |
| CS50-2 | 133.70926148080542 | 16.44629794176512 | 0.9996322433157738 | 127.9 | 0.29504335639885687 | 2.0649210194713623 | 0.6218495519444498 |
| CS50-3 | 125.3470456568155 | 16.801025216437317 | 0.9996794553225327 | 120.8 | 0.2948091516642137 | 2.0389806826272747 | 0.6190360604803296 |
| CD50-1 | 1593.8028809707662 | 123.60439897184588 | 0.9990234569128317 | 1584.5 | 0.7647517656956292 | 8.129164975773978 | 0.9762995566770363 |
| CD50-2 | 906.3517486347333 | 75.48745844237952 | 0.999639697843157 | 902.9 | 0.7497548893548297 | 7.361409897585875 | 0.9745535662288092 |
| CD50-3 | 1108.9310754603587 | 85.73649858831762 | 0.999363880329987 | 1101.1 | 0.7166618389443539 | 7.241671064306749 | 0.9630061203737016 |
| CS65-1 | 99.36221362229102 | 13.570363691340358 | 0.9997216976443696 | 95.1 | 0.3721121714251384 | 2.4451961934393083 | 0.6899415582637988 |
| CS65-2 | 154.22753896978034 | 19.944223814332577 | 0.9993315773779943 | 142.8 | 0.3322884470073832 | 2.378344296602948 | 0.6855412523562535 |
| CS65-3 | 73.90704764361885 | 12.798762914874228 | 0.9999527879932414 | 73.8 | 0.35978308744209386 | 2.2326413383175048 | 0.6735463038105466 |
| CD65-1 | 276.872041626894 | 30.054005184933267 | 0.9994409104462777 | 269.9 | 0.6285706852019675 | 5.0764779499041115 | 0.9367736800053024 |
| CD65-2 | 738.8625084052309 | 82.65147226022721 | 0.9977636417851107 | 709.0 | 0.5030982075855459 | 4.764145159525395 | 0.9115924265419959 |
| CD65-3 | 155.90553557558255 | 19.39815734388142 | 0.9995055163502634 | 148.9 | 0.48118814785409575 | 3.473246860271189 | 0.8670642205496476 |
