# Supplementary material for: Effects of the improved application of Bacillus halotolerans on the microbial community and volatile components of high-temperature daqu
Source: Front Microbiol. 2025 Jun 27;16:1626160. doi: 10.3389/fmicb.2025.1626160 (PMC12245780; doi:10.3389/fmicb.2025.1626160)
Supplement: Supplementary file 2 [file Table_2.doc]

Table S2. Alpha diversity indices of fungal metagenomes of samples.

| **Sample** | **Chao1** | **Goods_coverage** | **Observed_species** | **Pielou_e** | **Shannon** | **Simpson** |
| --- | --- | --- | --- | --- | --- | --- |
| CS0-1 | 275.1838053613054 | 0.9999194405954949 | 274.9 | 0.6001733788286467 | 4.863062423158587 | 0.9154947871920724 |
| CS0-2 | 88.92575757575757 | 0.9999709986143783 | 88.9 | 0.47217697216883503 | 3.0569209472414105 | 0.8229263910894808 |
| CS0-3 | 252.15793442668442 | 0.9999129958431346 | 251.8 | 0.535223896058051 | 4.269015275412147 | 0.8751581143089879 |
| CD0-1 | 175.18185104537403 | 0.9998034350530082 | 174.0 | 0.5197351934295338 | 3.8683596775000297 | 0.8589763240538698 |
| CD0-2 | 206.55416866415317 | 0.999851770695711 | 205.9 | 0.5232396428190309 | 4.021514796745717 | 0.8660262715357214 |
| CD0-3 | 226.61619883040936 | 0.9998421035671704 | 226.0 | 0.5437461735898731 | 4.252188310989181 | 0.8826504364211832 |
| CS3-1 | 212.3 | 0.9999613314858375 | 211.9 | 0.642090356070533 | 4.961586439834607 | 0.859049371779992 |
| CS3-2 | 207.175 | 0.9999258853478554 | 206.2 | 0.6209873298223079 | 4.774087841859949 | 0.8450694900548108 |
| CS3-3 | 195.2583333333333 | 0.9999516643572969 | 195.1 | 0.56089616196044 | 4.267329894109834 | 0.7715602110085398 |
| CD3-1 | 141.74731554284992 | 0.9994167499113846 | 133.2 | 0.4132805978273407 | 2.9166486287986144 | 0.7992825605228531 |
| CD3-2 | 160.95383596080745 | 0.99950375406825 | 156.6 | 0.40913522516113404 | 2.9829253856155753 | 0.8045953003238868 |
| CD3-3 | 173.27233879749429 | 0.9993587471401412 | 164.8 | 0.4118391644289889 | 3.0330032220313194 | 0.806247244676527 |
| CS8-1 | 308.0833333333333 | 0.9999548867334772 | 307.7 | 0.7606330202018797 | 6.286915005244385 | 0.9367251939224751 |
| CS8-2 | 289.12833333333333 | 0.9999323301002159 | 288.4 | 0.7258050304361531 | 5.9312219224495415 | 0.9181996844185468 |
| CS8-3 | 310.55 | 0.9999001063384139 | 308.5 | 0.7502866402342941 | 6.204211446031729 | 0.9314818829175652 |
| CD8-1 | 169.53333333333333 | 0.9999516643572971 | 168.8 | 0.6435391817403191 | 4.761653771780049 | 0.8661962366411347 |
| CD8-2 | 172.8 | 0.9999806657429187 | 172.7 | 0.6522562941663781 | 4.84764584324528 | 0.8762392755646446 |
| CD8-3 | 163.85 | 0.9999742209905584 | 163.7 | 0.6082705136824421 | 4.473770345292793 | 0.8479145121012873 |
| CS16-1 | 177.3 | 0.9999677762381978 | 177.0 | 0.9470564752670292 | 7.072244190946108 | 0.990847889474281 |
| CS16-2 | 176.61666666666667 | 0.9999387748525763 | 175.8 | 0.9465527177292943 | 7.059187412212459 | 0.9907824010877626 |
| CS16-3 | 177.0 | 0.9999967776238197 | 177.0 | 0.9399614053968002 | 7.019261007804959 | 0.9902511778424575 |
| CD16-1 | 273.451552569193 | 0.9990912899171848 | 263.8 | 0.48936069174387364 | 3.936046729970954 | 0.8560680596169685 |
| CD16-2 | 287.25847558358913 | 0.9989172816034545 | 274.4 | 0.45465256838299944 | 3.6827296195812487 | 0.8113075791665677 |
| CD16-3 | 256.98762868134213 | 0.9988302774465891 | 242.4 | 0.3808153704838032 | 3.0164848208338326 | 0.6854531687390834 |
| CS30-1 | 182.55 | 0.9999709986143781 | 182.4 | 0.7155910711881986 | 5.374768655459292 | 0.8765607904765703 |
| CS30-2 | 174.95 | 0.9999677762381978 | 174.8 | 0.6980383523370385 | 5.200078079538594 | 0.8564941409630282 |
| CS30-3 | 184.13333333333333 | 0.999935552476396 | 183.3 | 0.7238905366973759 | 5.442244219269929 | 0.8826073151652505 |
| CD30-1 | 138.0 | 0.9999548867334772 | 137.5 | 0.9368551868895848 | 6.654739669945886 | 0.9874589851961021 |
| CD30-2 | 134.9 | 0.9999871104952792 | 134.9 | 0.9437886920320052 | 6.678005420391074 | 0.9881013361331078 |
| CD30-3 | 140.9 | 0.9999742209905584 | 140.8 | 0.9383086796017045 | 6.697172076240301 | 0.98826036034049 |
| CS50-1 | 212.54650323568163 | 0.9995134211967904 | 207.2 | 0.28638062088194205 | 2.2036541936626337 | 0.5882816450733284 |
| CS50-2 | 89.21803252002555 | 0.9994457512970063 | 81.7 | 0.22274119955642752 | 1.4147420081860966 | 0.45513268458672423 |
| CS50-3 | 152.96423839970774 | 0.9994779750588083 | 146.1 | 0.24249204779949643 | 1.7436720980393943 | 0.5132013402513903 |
| CD50-1 | 165.18813288019172 | 0.999419972287565 | 156.5 | 0.2425646654207927 | 1.7682659913703642 | 0.3780118614739927 |
| CD50-2 | 58.84845238095238 | 0.9997518770341249 | 53.5 | 0.11499914242254057 | 0.660121294461606 | 0.13168813596818835 |
| CD50-3 | 107.32906356801092 | 0.9994618631779073 | 98.9 | 0.14508565826006442 | 0.9615599051089042 | 0.2006795678087554 |
| CS65-1 | 211.0253775637685 | 0.9995714239680342 | 206.7 | 0.2590943137078941 | 1.992790511450681 | 0.5914028868426391 |
| CS65-2 | 87.92354857732566 | 0.9994908645635292 | 80.3 | 0.22006565858058683 | 1.3922183071281917 | 0.5336927862309337 |
| CS65-3 | 127.62512974465149 | 0.9995295330776915 | 122.3 | 0.2264992860596226 | 1.5704955037142496 | 0.5510688138324251 |
| CD65-1 | 218.56361293315658 | 0.999439306544646 | 214.0 | 0.32157327107623634 | 2.489427190014651 | 0.7064171926488216 |
| CD65-2 | 96.07380477709425 | 0.999387748525763 | 87.4 | 0.2635239062781863 | 1.699384529472891 | 0.5710460621100534 |
| CD65-3 | 211.368658672339 | 0.9991009570457257 | 200.8 | 0.30483889082389454 | 2.3318632359439606 | 0.6872952959001157 |
