# Supplementary material for: Effects of the improved application of Bacillus halotolerans on the microbial community and volatile components of high-temperature daqu
Source: Front Microbiol. 2025 Jun 27;16:1626160. doi: 10.3389/fmicb.2025.1626160 (PMC12245780; doi:10.3389/fmicb.2025.1626160)
Supplement: Supplementary file 3 [file Table_3.doc]

**Table S3. Detection results of volatile components (VOCs) in 14 different samples during cultivation.**

|  | Content / (mg/L) | | | | | | | | | | | | | | RI |
| --- | --- | --- | --- | --- | --- | --- | --- | --- | --- | --- | --- | --- | --- | --- | --- |
|  | CD0 | CS0 | CD3 | CS3 | CD8 | CS8 | CD16 | CS16 | CD30 | CS30 | CD50 | CS50 | CD65 | CS65 |  |
| **Alcohols** |  |  |  |  |  |  |  |  |  |  |  |  |  |  |  |
| (2R,3R)-(-)-2, 3-Butanediol | 0.001±0 | 0.001±0 | 0.012±0 | 0.014±0 | 0.032±0 | 0.017±0.001 | 0.049±0 | 0.016±0 | n.d. | n.d. | 0.013±0 | n.d. | n.d. | 0.041±0.001 | 784 |
| (2S,3S)-(+)-2, 3-Butanediol | n.d. | n.d. | n.d. | n.d. | n.d. | n.d. | n.d. | n.d. | n.d. | 0.032±0 | 0.008±0.006 | 0.008±0 | 0.003±0 | 0.021±0 | 793 |
| 2,3-butanediol | 0.001±0 | n.d. | 0.003±0 | n.d. | n.d. | n.d. | 0.001±0 | n.d. | 0.026±0.001 | n.d. | n.d. | 0.009±0 | n.d. | n.d. | 834 |
| Hexanol | 0.024±0.001 | 0.035±0.001 | n.d. | 0.142±0.001 | n.d. | n.d. | n.d. | 0.031±0 | n.d. | n.d. | n.d. | n.d. | n.d. | n.d. | 864 |
| 1-heptanol | 0.002±0 | 0.002±0 | 0.004±0 | 0.001±0 | 0.005±0 | n.d. | n.d. | n.d. | n.d. | n.d. | n.d. | n.d. | n.d. | n.d. | 964 |
| 1-octen-3-ol | 0.003±0 | 0.003±0 | 0.008±0 | 0.002±0 | 0.011±0 | n.d. | n.d. | n.d. | n.d. | n.d. | n.d. | n.d. | n.d. | n.d. | 973 |
| 2-ethylhexanol | 0.004±0 | 0.005±0 | n.d. | n.d. | n.d. | 0.016±0 | n.d. | n.d. | 0.007±0.001 | n.d. | 0.001±0 | n.d. | n.d. | n.d. | 1021 |
| 2-octene-1-ol | n.d. | n.d. | 0.004±0 | 0.007±0 | 0.007±0 | n.d. | n.d. | n.d. | n.d. | n.d. | n.d. | n.d. | n.d. | n.d. | 1061 |
| 1-Octanol | 0.002±0 | 0.001±0 | 0.003±0 | 0.007±0 | 0.005±0 | 0.005±0 | n.d. | n.d. | n.d. | n.d. | n.d. | n.d. | n.d. | n.d. | 1064 |
| **Acids** |  |  |  |  |  |  |  |  |  |  |  |  |  |  |  |
| acetic acid | 0.002±0 | 0.005±0 | 0.296±0.002 | 0.323±0.004 | 0.377±0.003 | n.d. | 0.005±0 | 0.364±0.002 | n.d. | n.d. | n.d. | 0.006±0 | 0.055±0 | 0.046±0.001 | 744 |
| 2-methylbutyrate | n.d. | n.d. | n.d. | n.d. | n.d. | 0.069±0 | n.d. | n.d. | n.d. | 0.031±0 | 0.050±0.002 | 0.009±0 | 0.070±0.001 | 0.035±0.001 | 894 |
| Valproic acid | n.d. | n.d. | n.d. | n.d. | n.d. | 0.001±0 | n.d. | 0.002±0 | 0.007±0 | 0.004±0 | 0.001±0 | 0.001±0 | 0.086±0.001 | 0.002±0 | 1030 |
| Octanoic acid | n.d. | n.d. | 0.004±0 | 0.005±0 | 0.003±0 | n.d. | 0.007±0 | 0.008±0 | n.d. | n.d. | 0.002±0 | 0.002±0 | 0.006±0 | 0.003±0 | 1162 |
| Pentanoicacid, 2-hydroxy-4-met | n.d. | n.d. | n.d. | n.d. | n.d. | n.d. | n.d. | n.d. | 0.035±0.002 | 0.010±0.001 | n.d. | 0.002±0 | n.d. | 0.001±0 | 1169 |
| 2-methyl-3-hydroxy-2,2,4-trimethylpentylpropionic acid | 0.001±0 | 0.004±0 | 0.002±0 | 0.002±0 | 0.001±0 | 0.002±0 | 0.001±0 | 0.001±0 | 0.001±0 | n.d. | n.d. | n.d. | n.d. | n.d. | 1358 |
| **Esters** |  |  |  |  |  |  |  |  |  |  |  |  |  |  |  |
| Ethyl Butyrate | 0.008±0 | n.d. | n.d. | n.d. | n.d. | n.d. | n.d. | n.d. | n.d. | n.d. | n.d. | n.d. | n.d. | n.d. | 798 |
| butyl acetate | n.d. | n.d. | 0.018±0 | 0.029±0 | n.d. | 0.028±0.001 | n.d. | n.d. | n.d. | n.d. | n.d. | n.d. | n.d. | n.d. | 811 |
| ethyl valerate | 0.008±0 | 0.007±0 | n.d. | 0.015±0 | n.d. | n.d. | n.d. | n.d. | n.d. | n.d. | n.d. | n.d. | n.d. | n.d. | 894 |
| ethyl caproate | n.d. | n.d. | 0.047±0 | 0.057±0 | 0.036±0 | 0.035±0 | n.d. | n.d. | n.d. | n.d. | n.d. | n.d. | n.d. | n.d. | 991 |
| 2-Hydroxy-4-methylpentanoic acid ethyl ester | n.d. | n.d. | 0.006±0 | 0.008±0 | 0.018±0.001 | n.d. | 0.007±0 | 0.017±0 | 0.011±0.001 | 0.031±0.001 | 0.004±0 | 0.008±0 | 0.002±0 | n.d. | 1051 |
| isoamyl lactate | n.d. | n.d. | n.d. | n.d. | n.d. | n.d. | n.d. | n.d. | n.d. | 0.009±0 | n.d. | 0.002±0 | n.d. | 0.002±0 | 1061 |
| Propyl caproate | n.d. | n.d. | n.d. | n.d. | n.d. | n.d. | n.d. | n.d. | 0.003±0 | 0.003±0 | n.d. | n.d. | n.d. | n.d. | 1085 |
| ethyl enanthate | 0.005±0 | 0.010±0 | 0.014±0 | 0.012±0 | 0.004±0 | 0.033±0 | n.d. | n.d. | 0.007±0 | 0.005±0 | n.d. | 0.001±0 | n.d. | 0.001±0 | 1087 |
| Diisopropyl adipate | n.d. | n.d. | n.d. | 0.001±0 | 0.001±0 | n.d. | n.d. | 0.003±0 | 0.001±0 | 0.005±0 | n.d. | 0.001±0 | n.d. | 0.001±0 | 1166 |
| Ethyl octanoate | n.d. | n.d. | 0.001±0 | 0.001±0 | 0.001±0 | 0.001±0 | n.d. | n.d. | 0.003±0 | 0.003±0 | n.d. | n.d. | n.d. | 0.002±0 | 1182 |
| Ethyl succinate | n.d. | n.d. | n.d. | n.d. | n.d. | n.d. | n.d. | 0.002±0.002 | 0.002±0 | 0.001±0 | n.d. | n.d. | n.d. | n.d. | 1289 |
| 2,2,4-trimethyl-1,3-pentanedio | n.d. | n.d. | n.d. | n.d. | n.d. | 0.001±0 | n.d. | n.d. | n.d. | 0.001±0 | n.d. | n.d. | n.d. | n.d. | 1334 |
| Ethyl Palmitate | n.d. | n.d. | 0.001±0 | 0.007±0 | 0.002±0 | 0.005±0 | 0.006±0 | 0.007±0 | n.d. | 0.001±0 | n.d. | n.d. | 0.002±0 | 0.002±0 | 1966 |
| ethyl oleate | 0.003±0 | 0.005±0 | n.d. | 0.011±0 | 0.003±0 | 0.009±0 | 0.005±0 | 0.006±0 | n.d. | n.d. | n.d. | n.d. | n.d. | n.d. | 2139 |
| **Aldehydes and ketones** |  |  |  |  |  |  |  |  |  |  |  |  |  |  |  |
| 3-hydroxy-2-butanone | n.d. | n.d. | 0.002±0 | 0.001±0 | 0.006±0 | 0.005±0 | 0.003±0 | 0.003±0 | 0.002±0 | 0.003±0 | n.d. | 0.001±0 | 0.020±0.001 | n.d. | 794 |
| 2-Acetone | n.d. | n.d. | 0.003±0 | 0.003±0 | n.d. | 0.004±0 | n.d. | n.d. | n.d. | n.d. | n.d. | n.d. | n.d. | n.d. | 883 |
| 3-octen-2-one | n.d. | n.d. | 0.001±0 | 0.002±0 | 0.006±0.008 | n.d. | n.d. | n.d. | n.d. | n.d. | n.d. | n.d. | n.d. | n.d. | 1031 |
| nonanal | 0.004±0 | 0.001±0 | 0.005±0 | 0.011±0 | 0.010±0 | 0.010±0 | 0.010±0 | 0.013±0 | 0.015±0.001 | n.d. | 0.002±0 | 0.002±0 | n.d. | 0.001±0 | 1093 |
| Decanal | n.d. | n.d. | 0.001±0 | 0.001±0 | n.d. | 0.001±0 | 0.001±0 | n.d. | n.d. | n.d. | 0.002±0 | 0.002±0 | n.d. | n.d. | 1192 |
| **Aromatic and phenolic compounds** |  |  |  |  |  |  |  |  |  |  |  |  |  |  |  |
| Methoxyphenyl oxime | 0.027±0 | 0.020±0 | 0.031±0 | 0.027±0 | 0.038±0 | n.d. | 0.024±0 | 0.019±0 | n.d. | n.d. | n.d. | n.d. | n.d. | n.d. | 916 |
| benzaldehyde | 0.004±0 | 0.007±0 | 0.062±0 | 0.182±0.002 | 0.324±0.003 | 0.180±0.010 | 0.126±0.002 | 0.434±0.003 | 0.165±0.010 | 0.361±0.009 | 0.124±0.004 | 0.098±0.001 | 0.111±0.003 | 0.051±0 | 952 |
| Benzyl alcohol | n.d. | n.d. | n.d. | 0.015±0 | n.d. | n.d. | n.d. | 0.004±0 | 0.004±0 | 0.004±0 | 0.001±0 | 0.001±0 | 0.007±0 | 0.007±0 | 1029 |
| phenylacetaldehyde | n.d. | n.d. | 0.029±0 | 0.032±0.001 | 0.045±0 | 0.054±0 | 0.039±0 | 0.031±0 | 0.124±0.005 | 0.022±0 | 0.033±0.001 | 0.006±0 | 0.048±0 | 0.021±0 | 1037 |
| acetophenone | 0.001±0 | 0.001±0 | 0.001±0 | 0.002±0 | 0.003±0 | n.d. | 0.010±0 | 0.006±0 | n.d. | 0.013±0 | 0.003±0 | 0.004±0 | 0.019±0 | 0.005±0 | 1058 |
| Methyl benzoate | n.d. | n.d. | n.d. | 0.002±0 | 0.004±0 | n.d. | 0.008±0 | 0.008±0 | n.d. | 0.004±0 | n.d. | 0.001±0 | n.d. | 0.013±0 | 1084 |
| Phenylethanol | 0.003±0 | n.d. | n.d. | n.d. | 0.011±0 | 0.044±0 | 0.033±0 | 0.055±0 | 0.084±0 | 0.049±0.001 | 0.043±0.056 | 0.013±0 | 0.040±0.001 | 0.044±0.001 | 1102 |
| benzyl methyl ketone | n.d. | 0.000±0.001 | n.d. | n.d. | 0.001±0 | 0.002±0 | 0.002±0 | 0.003±0 | 0.005±0 | 0.007±0 | 0.001±0 | 0.002±0 | 0.005±0 | 0.002±0 | 1115 |
| Ortho phenyldimethyl ether | n.d. | n.d. | 0.002±0 | 0.006±0 | 0.006±0 | 0.025±0 | 0.006±0 | 0.013±0 | 0.002±0 | 0.003±0 | 0.001±0 | 0.001±0 | 0.006±0 | 0.004±0 | 1132 |
| ethyl benzoate | 0.001±0 | n.d. | 0.002±0 | 0.002±0 | 0.002±0 | 0.003±0 | n.d. | 0.002±0 | n.d. | n.d. | n.d. | n.d. | n.d. | n.d. | 1160 |
| naphthalene | 0.001±0 | 0.002±0 | 0.001±0 | 0.001±0 | 0.001±0 | 0.001±0 | n.d. | n.d. | n.d. | n.d. | n.d. | n.d. | n.d. | n.d. | 1175 |
| 3,4-Dimethylbenzaldehyde | n.d. | n.d. | 0.005±0 | 0.004±0 | 0.007±0 | 0.001±0 | 0.002±0 | 0.003±0 | n.d. | n.d. | n.d. | 0.001±0 | n.d. | 0.003±0 | 1202 |
| Ethyl phenylacetate | n.d. | n.d. | 0.002±0 | 0.010±0 | 0.005±0 | 0.006±0 | 0.003±0 | 0.004±0 | 0.018±0.001 | 0.009±0 | 0.002±0 | 0.002±0 | 0.004±0 | 0.002±0 | 1228 |
| β-Ethylphenylethanol | n.d. | n.d. | n.d. | n.d. | n.d. | n.d. | n.d. | n.d. | 0.001±0 | 0.003±0 | 0.002±0 | 0.001±0 | 0.002±0 | 0.002±0 | 1238 |
| Phenylethyl Acetate | n.d. | n.d. | 0.006±0 | 0.009±0 | 0.011±0 | 0.003±0 | 0.004±0 | 0.004±0 | 0.002±0 | 0.008±0 | n.d. | 0.002±0 | n.d. | 0.003±0 | 1241 |
| 2-phenylbutanal | n.d. | n.d. | n.d. | 0.004±0 | 0.014±0 | 0.014±0 | 0.011±0 | 0.030±0 | 0.028±0.001 | 0.094±0.001 | 0.014±0.001 | 0.023±0.001 | 0.008±0 | 0.003±0 | 1256 |
| 2-methoxy-4-vinylphenol | n.d. | n.d. | 0.001±0 | 0.001±0 | 0.002±0 | 0.004±0 | 0.002±0 | n.d. | n.d. | n.d. | n.d. | n.d. | n.d. | n.d. | 1295 |
| Ethyl phenylpropionate | n.d. | n.d. | 0.001±0 | 0.001±0 | 0.002±0 | n.d. | n.d. | n.d. | 0.006±0 | 0.004±0 | n.d. | n.d. | 0.021±0 | n.d. | 1333 |
| 3,4-dimethoxystyrene | n.d. | n.d. | n.d. | 0.002±0 | 0.001±0 | 0.002±0 | n.d. | n.d. | n.d. | n.d. | n.d. | n.d. | n.d. | n.d. | 1347 |
| 4-methyl-2-phenyl-2-pentenal | n.d. | n.d. | n.d. | n.d. | n.d. | 0.004±0 | n.d. | 0.002±0 | 0.003±0 | 0.006±0 | 0.012±0 | 0.016±0.001 | 0.008±0 | n.d. | 1351 |
| Vanillin | n.d. | n.d. | n.d. | n.d. | n.d. | 0.001±0 | n.d. | 0.003±0 | 0.001±0 | n.d. | n.d. | n.d. | 0.002±0 | n.d. | 1380 |
| Cocal | n.d. | n.d. | 0.001±0 | 0.002±0 | 0.004±0 | 0.004±0 | 0.008±0 | 0.007±0 | 0.014±0 | 0.019±0 | 0.024±0 | 0.045±0.001 | 0.028±0.001 | 0.026±0.001 | 1441 |
| 2,4-di-tert-butylphenol | 0.001±0 | 0.001±0 | 0.007±0 | 0.003±0 | 0.005±0 | 0.002±0 | 0.001±0 | 0.002±0 | 0.006±0 | 0.004±0 | 0.002±0 | 0.001±0 | 0.005±0 | 0.002±0 | 1486 |
| Furan |  |  |  |  |  |  |  |  |  |  |  |  |  |  |  |
| furfural | n.d. | n.d. | 0.011±0 | n.d. | 0.046±0.001 | 0.044±0 | n.d. | 0.053±0.001 | 0.052±0 | 0.283±0.016 | n.d. | 0.078±0.001 | 0.137±0.002 | n.d. | 824 |
| 3-Furfural | n.d. | n.d. | 0.011±0.014 | 0.032±0 | 0.050±0 | 0.060±0 | 0.092±0 | 0.071±0 | 0.057±0.002 | n.d. | 0.064±0.001 | n.d. | n.d. | n.d. | 825 |
| 2-Furoxaldehyde ethanol | n.d. | n.d. | 0.002±0 | 0.018±0.024 | 0.008±0 | n.d. | n.d. | n.d. | n.d. | n.d. | n.d. | n.d. | 0.002±0 | n.d. | 846 |
| Ethyl 2-furanate, ethyl 2-furancarboxylate | n.d. | n.d. | n.d. | 0.001±0 | 0.001±0 | n.d. | n.d. | 0.003±0 | n.d. | n.d. | n.d. | n.d. | n.d. | n.d. | 1044 |
| 3-phenylfuran | n.d. | n.d. | n.d. | n.d. | n.d. | 0.001±0 | 0.001±0 | 0.001±0 | 0.001±0 | 0.001±0 | 0.001±0 | n.d. | 0.001±0 | n.d. | 1210 |
| 2-pentacylfuran | n.d. | n.d. | n.d. | n.d. | n.d. | n.d. | n.d. | n.d. | n.d. | 0.001±0 | 0.001±0 | n.d. | n.d. | n.d. | 1251 |
| 2-phenyl-3- (2-furanyl) acrolein | n.d. | n.d. | n.d. | n.d. | n.d. | n.d. | n.d. | n.d. | n.d. | 0.005±0 | 0.001±0 | 0.001±0 | 0.003±0 | 0.003±0 | 1662 |
| **Others** |  |  |  |  |  |  |  |  |  |  |  |  |  |  |  |
| 2,3-dimethylpyrazine | n.d. | n.d. | n.d. | n.d. | n.d. | 0.035±0.001 | n.d. | n.d. | n.d. | n.d. | n.d. | n.d. | n.d. | n.d. | 910 |
| butyrolactone | n.d. | n.d. | n.d. | 0.001±0 | 0.001±0 | n.d. | n.d. | n.d. | n.d. | n.d. | n.d. | n.d. | n.d. | n.d. | 921 |
| 2,5-dimethylpyrazine | n.d. | n.d. | n.d. | n.d. | 0.007±0 | n.d. | n.d. | n.d. | n.d. | n.d. | n.d. | n.d. | n.d. | n.d. | 926 |
| Trimethylpyrazine | n.d. | n.d. | n.d. | n.d. | n.d. | 0.161±0.001 | 0.116±0.001 | 0.103±0.007 | n.d. | 0.017±0.001 | n.d. | n.d. | 0.045±0.002 | n.d. | 992 |
| 2-methyl-5-isopropylpyrazine | n.d. | n.d. | n.d. | n.d. | n.d. | n.d. | n.d. | 0.001±0 | n.d. | n.d. | n.d. | n.d. | 0.004±0 | 0.001±0 | 1039 |
| 2-methyl-3-isopropylpyrazine | n.d. | n.d. | n.d. | n.d. | n.d. | n.d. | n.d. | n.d. | n.d. | n.d. | 0.004±0 | n.d. | 0.004±0 | 0.001±0 | 1047 |
| 2,6-diethylpyrazine | n.d. | n.d. | 0.003±0 | n.d. | n.d. | n.d. | n.d. | n.d. | n.d. | n.d. | 0.001±0 | n.d. | n.d. | n.d. | 1067 |
| Tetramethylpyrazine | 0.014±0 | n.d. | 0.005±0 | 0.007±0 | 0.008±0 | 0.013±0 | n.d. | 0.003±0 | 0.005±0 | 0.008±0 | 0.005±0 | 0.006±0 | 0.005±0 | 0.006±0 | 1076 |
| 2,3-dimethyl-5-ethylpyrazine | n.d. | n.d. | 0.003±0 | n.d. | n.d. | n.d. | n.d. | n.d. | n.d. | n.d. | n.d. | n.d. | n.d. | n.d. | 1077 |
| 2,3-dimethyl-5-n-propylpyrazine | n.d. | n.d. | n.d. | n.d. | n.d. | n.d. | n.d. | 0.001±0 | n.d. | 0.002±0 | n.d. | 0.001±0 | n.d. | n.d. | 1120 |
| 2,5-dimethyl-3-isopropylpyrazine | n.d. | n.d. | n.d. | n.d. | n.d. | 0.013±0 | 0.008±0 | n.d. | n.d. | n.d. | n.d. | n.d. | n.d. | n.d. | 1120 |
| 2,3,5-trimethyl-6-ethylpyrazine | n.d. | n.d. | n.d. | n.d. | n.d. | n.d. | n.d. | n.d. | n.d. | n.d. | 0.001±0 | n.d. | 0.002±0 | n.d. | 1146 |
| 2,3,5-trimethyl-6-propylpyrazine | n.d. | n.d. | n.d. | n.d. | n.d. | 0.001±0 | 0.002±0 | n.d. | n.d. | n.d. | n.d. | n.d. | n.d. | n.d. | 1168 |
| 2-Isopentyl-6-methylpyrazine | n.d. | n.d. | n.d. | n.d. | n.d. | n.d. | n.d. | n.d. | n.d. | n.d. | 0.001±0 | n.d. | 0.004±0 | n.d. | 1237 |
| 2-Isobutyl-3,5,6-trimethylpyrazine | n.d. | n.d. | n.d. | n.d. | n.d. | 0.003±0 | 0.010±0 | n.d. | n.d. | n.d. | n.d. | n.d. | n.d. | n.d. | 1261 |
| 2,3-dimethyl-5-isopentylpyrazine | n.d. | n.d. | n.d. | n.d. | n.d. | 0.001±0 | 0.001±0 | n.d. | n.d. | n.d. | n.d. | n.d. | 0.001±0 | n.d. | 1321 |
| Gamma nonanolactone | 0.003±0 | 0.002±0 | n.d. | 0.009±0 | n.d. | 0.008±0 | 0.007±0 | 0.006±0 | 0.003±0 | 0.010±0.001 | 0.003±0 | 0.003±0 | 0.007±0 | 0.005±0 | 1345 |
