# Supplementary material for: Effects of the improved application of Bacillus halotolerans on the microbial community and volatile components of high-temperature daqu
Source: Front Microbiol. 2025 Jun 27;16:1626160. doi: 10.3389/fmicb.2025.1626160 (PMC12245780; doi:10.3389/fmicb.2025.1626160)
Supplement: Supplementary file 4 [file Table_4.docx]

**Table S4**. The RDA scores of dominant microbial genus with physicochemical parameters and VOCs.

| Category | | | Microbial genus | | RDA1 | RDA2 | | | |  |
| --- | --- | --- | --- | --- | --- | --- | --- | --- | --- | --- |
| The RDA scores of physicochemical parameters and bacterial genera | | | *Other* | | 0.957844820678076 | -0.157178407 | | | |  |
|  |  |  | *Sphingomonas* | | 0.0970434876731054 | -0.100609953 | | | |  |
|  |  |  | *Pseudonocardiaceae* | | -0.071134761 | -0.204427532 | | | |  |
|  |  |  | *Thermoactinomyces* | | -0.07443746 | -0.248297291 | | | |  |
|  |  |  | *Weissella* | | -0.02274982 | 0.333485462236213 | | | |  |
|  |  |  | *Acetobacter* | | 0.105777579345952 | 0.149771115687575 | | | |  |
|  |  |  | *Bacillus* | | 0.0267855703865218 | -0.598666137 | | | |  |
|  |  |  | *Saccharopolyspora* | | -0.190812096 | -0.553152182 | | | |  |
|  |  |  | *Lactobacillus* | | 0.116968363115985 | 0.252404447628066 | | | |  |
| The RDA scores of physicochemical parameters and fungal genera | | | *Other* | | 0.231392191684298 | -0.686414346 | | | |  |
|  |  |  | *Mortierella* | | 0.013249293918867 | -0.040603935 | | | |  |
|  |  |  | *Fusarium* | | 0.0222834102282718 | -0.031535505 | | | |  |
|  |  |  | *Cladosporium* | | 0.0336819737075965 | -0.115027369 | | | |  |
|  |  |  | *Millerozyma* | | 0.0324453743713161 | -0.124788569 | | | |  |
|  |  |  | *Aspergillus* | | 0.0415038970995589 | -0.072379792 | | | |  |
|  |  |  | *Alternaria* | | 0.19106544354021 | 0.0947380517211425 | | | |  |
|  |  |  | *Rhizomucor* | | -0.042298446 | 0.38550079277791 | | | |  |
|  |  |  | *Blumeria* | | 0.533718837801832 | 0.513305170345356 | | | |  |
|  |  |  | *Thermomyces* | | -0.35523506 | -0.145925268 | | | |  |
|  |  |  | *Thermoascus* | | -0.701806882 | 0.223130723840447 | | | |  |
| The RDA scores of VOCs and bacterial genera | | | *Other* | | 0.926276153566032 | | -0.21916635 | | | |
|  |  |  | *Sphingomonas* | | 0.0995378203108661 | | 0.0136120416063265 | | | |
|  |  |  | *Pseudonocardiaceae* | | -0.014960057 | | 0.133667075044534 | | | |
|  |  |  | *Thermoactinomyces* | | 0.0153277724285169 | | 0.160899516236673 | | | |
|  |  |  | *Weissella* | | 0.0398049647964955 | | -0.089252161 | | | |
|  |  |  | *Acetobacter* | | -0.199379369 | | -0.66906891 | | | |
|  |  |  | *Bacillus* | | 0.282396746637012 | | 0.109909194305976 | | | |
|  |  |  | *Saccharopolyspora* | | -0.036243473 | | 0.362581550996326 | | | |
|  |  |  | *Lactobacillus* | | -0.096165929 | | -0.555752387 | | | |
| The RDA scores of VOCs and fungal genera | *Other* | | 0.764604018952871 | | | | -0.109321337 |  |  |  |
|  | *Mortierella* | | 0.0480004419111641 | | | | -0.013847799 |  |  |  |
|  | *Fusarium* | | 0.0574240898868277 | | | | -0.004654864 |  |  |  |
|  | *Cladosporium* | | 0.0574907270217625 | | | | -0.057980279 |  |  |  |
|  | *Millerozyma* | | 0.120643134014797 | | | | -0.127368605 |  |  |  |
|  | *Aspergillus* | | 0.0842992970597633 | | | | 0.0342549571158335 |  |  |  |
|  | *Alternaria* | | -0.141738854 | | | | 0.0101189754009018 |  |  |  |
|  | *Rhizomucor* | | -0.033682871 | | | | 0.714763745464394 |  |  |  |
|  | *Blumeria* | | -0.431858967 | | | | 0.298625851506587 |  |  |  |
